# Reinterpreting the Frequency Dependence of Cortical Auditory-Evoked Response Amplitudes in Light of Current Understanding of Cortical Tonotopic Organization

**DOI:** 10.1177/23312165261454531

**Published:** 2026-08-04

**Authors:** Carl Rushworth, Alexander J. Hardy, Magdalena Sereda, Ben J. Gurer, Julien Besle, Susan T. Francis, Rebecca S. Dewey, Denis Schluppeck, Justin M. Ales, Vassilis Pelekanos, Michael A. Akeroyd, Katrin Krumbholz

**Affiliations:** 1 Hearing Sciences, 170718 School of Medicine, University of Nottingham, Nottingham, UK; 2 NIHR Nottingham Biomedical Research Centre, Hearing Theme, Nottingham, UK; 3 School of Psychology, 270394 Nottingham Trent University, Nottingham, UK; 4 School of Psychology, University of Plymouth, Plymouth, UK; 5 Sir Peter Mansfield Imaging Centre, 642994 University of Nottingham, Nottingham, UK; 6 School of Psychology, University of Nottingham, Nottingham, UK; 7 School of Psychology and Neuroscience, 105641 University of St Andrews, St Andrews, UK

**Keywords:** cortical auditory-evoked potentials/fields, tonotopic mapping, intra-cortical myelination, functional parcellation, EEG forward modeling, fMRI–EEG fusion

## Abstract

Tonotopy is a fundamental feature of auditory cortical organization, yet its influence on cortical auditory-evoked responses (AERs) remains unclear. Consequently, key properties of cortical AERs—such as their marked amplitude reduction with increasing stimulus frequency—still lack a coherent mechanistic explanation. To address this gap, we combined a meta-analysis of frequency-specific AER amplitudes with forward simulations of AERs informed by current knowledge of auditory cortical tonotopic layout and functional organization. The meta-analysis used a semi-systematic search covering all known automatic cortical AER components—both transient-evoked and steady-state—along with selected subcortical components for comparison. Forward simulations were based on a functional parcellation of the human supratemporal auditory region into subdivisions forming distinct tonotopic maps, and an idealized model of each division's intrinsic tonotopic layout. Parcellation was achieved using a novel, largely automated procedure applied to high-field (3 T) and ultra-high-field (7 T) functional and microstructural MRI mapping data from 30 individual hemispheres. Meta-analytic results revealed that, whilst all cortical AER components consistently show frequency-related amplitude reduction, reduction is greater in steady-state compared to transient-evoked components. Simulations indicated that frequency-related amplitude reduction arising from cortical morphology is confined to the highly myelinated central portion of Heschl's gyrus, suggesting that differences in reduction amount between steady-state and transient-evoked components may reflect differences in the relative strengths of their primary contributions. Our findings provide a new perspective on cortical AER generation. They represent an important step toward explaining morphology-related variability in AER amplitudes and establishing a quantitative link to underlying source strengths.

## Introduction

Auditory-evoked electro- and magnetoencephalographic (EEG/MEG) responses, hereafter referred to as auditory-evoked responses or AERs, represent a direct measure of auditory neural activity and can be recorded with essentially unlimited temporal resolution. Particularly when measured with EEG, AERs enable tracking of neural activity across the entire auditory processing hierarchy—from the auditory nerve to cortex (e.g., Holt & Ozdamar, 2016). AERs thus hold considerable promise for revealing how auditory information is propagated and transformed across processing levels, and how these transformations may be altered by experience, hearing loss, or aging (Auerbach & Gritton, 2022; Gold & Bajo, 2014; Recanzone, 2017). Quantitative interpretation of AERs, however, remains limited by poor understanding of their relationship to underlying neuroelectric sources. The scalp characteristics of AERs—such as spatial distribution and amplitude—are determined not only by the strengths of these sources but also by their geometric configurations.

Cortical AERs reflect the aggregate activity of thousands, or even millions, of microscopic electrical currents, each oriented perpendicular to the local cortical surface (Luck, 2014). Depending on the morphology (shape and orientation) of the response-generating surface patch(es), these currents can either reinforce or cancel one another, creating scalp responses with widely varying amplitudes and scalp distributions (or “scalp topographies”), even when underlying sources are equally strong and originate from the same functional area(s) (Scherg et al., 2019). A particularly striking example of the effect of surface morphology can be seen in the visual domain: EEG responses from the primary visual area, V1, can exhibit opposite scalp polarities for stimuli in the upper versus lower visual hemifields, because, in the V1 retinotopic map, these hemifields are represented on opposite banks of the calcarine fissure (Ales et al., 2010b).

The auditory-responsive supratemporal region is the principal contributor to the common obligatory (or “exogenous”; Picton, 1988) components of cortical AERs, as measured with either MEG or with the vertical montage in EEG (between the vertex and mastoid channels; Scherg & von Cramon, 1985, 1986). Much, if not all, of this region is tonotopically organized (e.g., Moerel et al., 2012; Striem-Amit et al., 2011). By analogy to the effect of retinotopy on visual-evoked responses, morphology-related variation in obligatory cortical AERs should therefore be primarily dictated by the supratemporal tonotopic layout. Although the supratemporal auditory surface is comparatively flatter than the visual cortical surface, and so, morphology-related variation in AERs should be more modest, the transverse gyrification of the supratemporal plane (STP) through Heschl's gyrus (HG), which, in individual subjects or hemispheres, can be partially or fully duplicated, or—in rare cases—even triplicated (Marie et al., 2015), could exert substantial impact on frequency-related and inter-individual variation in AER characteristics (amplitude and scalp topography). Understanding morphology-related effects on cortical AERs may thus help to account for a substantial proportion of their inter-individual variation and establish a more direct quantitative relationship between measured AER amplitudes and underlying source strengths.

**Figure 1.**
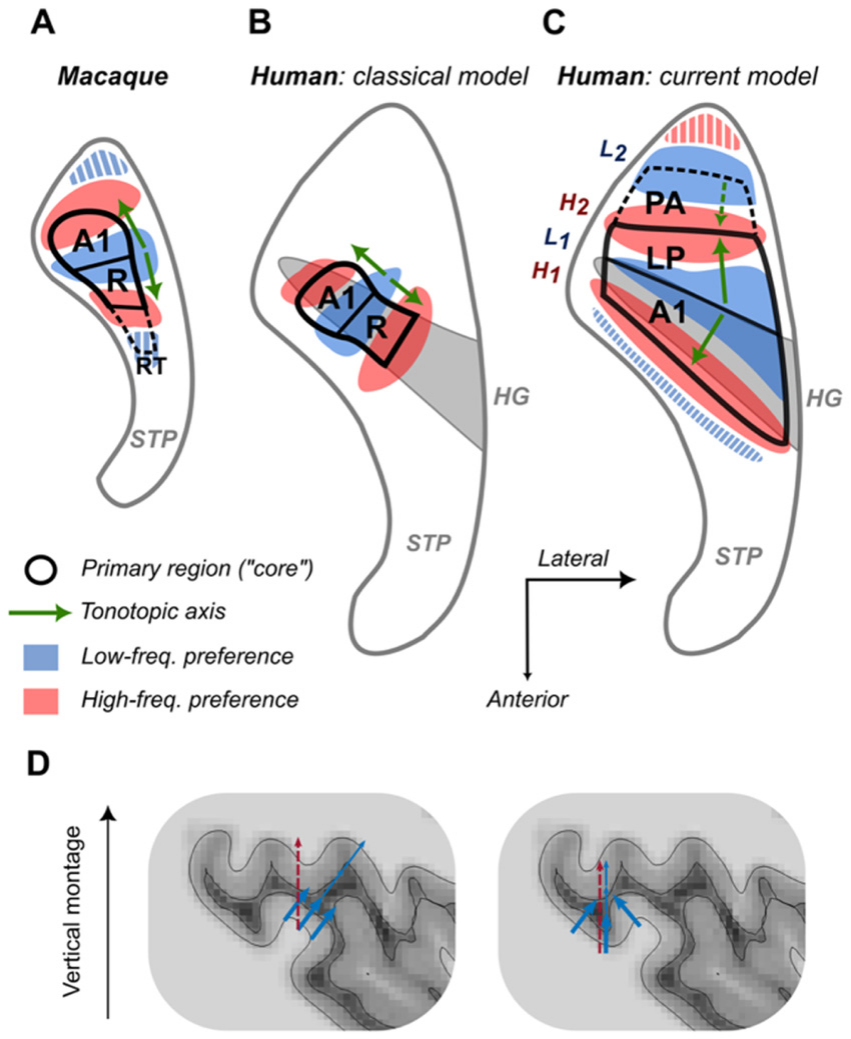
Tonotopic layout of macaque and human auditory cortex and effect on aggregate AER amplitude. (A) In macaques, regions of low- and high-frequency preference—representing tonotopic map endpoints (blue and red highlight)—extend across the width of the supratemporal plane (STP), indicating that the predominant tonotopic axis (direction of tonotopic gradients; green arrows) runs along the STP length (Petkov et al., 2006 and references therein). In humans (B&C), early evidence (B; “classical model”), primarily from magnetoencephalography (MEG), suggested that the human tonotopic axis is rotated compared to macaques, to run parallel to the long axis of Heschl's gyrus (HG), which, in humans, is the site of the primary auditory region (black outlines). More recent evidence—from functional and microstructural MRI—has suggested that the human tonotopic axis runs roughly perpendicular to the HG long axis, and has also called into doubt the strict homology between the human and macaque models (Besle et al., 2019). Hatched blue and red highlight: regions where frequency preference is not fully confirmed. (D) Cortical surface morphology can affect the measured scalp amplitude of cortical auditory-evoked responses (AERs) by (a) altering the net orientation of the response-generating cortical patch relative to the vertical EEG montage (left panel), and (b) introducing dispersion in microscopic current orientations due to local surface curvature within the response-generating patch, leading to partial cancelation in the aggregate response (right panel). Bold blue arrows: elemental currents; thin blue arrows: aggregate current; red dashed arrows: current component projected onto the vertical montage.

In the visual domain, research on morphology-related variation in evoked responses has been critically enabled by information on visual cortical functional organization—areal parcellation and retinotopic layout—obtained primarily through functional magnetic resonance imaging (fMRI; e.g., Ales et al., 2010a; Hagler et al., 2009; Inverso et al., 2016; Nasiotis et al., 2017). Early efforts at characterizing the functional organization (tonotopic layout) of the auditory cortex in humans—using positron emission tomography (PET), single-photon emission tomography (SPECT), EEG, fMRI, and, most frequently, MEG—often produced inconsistent or inconclusive results that have largely failed to replicate in later studies (reviewed in Johnsrude et al., 2002; Lütkenhöner et al., 2003b; Saenz & Langers, 2014). At the time, the prevailing consensus was that the predominant tonotopic axis—the main direction of change in local cortical frequency preference—ran parallel to the long axis of HG (Figure 1B), which post-mortem anatomical studies had identified as the likely site of the primary auditory region (reviewed in Abdul-Kareem & Sluming, 2008; Baumann et al., 2013). This suggested that the human auditory cortex organization is homologous to that in non-human primates, specifically, macaques, but rotated relative to it (Figure 1A). In macaques, the STP lacks a distinct HG, and the predominant tonotopic axis runs anteroposteriorly along its length, parallel to the long axis of a central primary region, referred to as “core” (Hackett et al., 2001; Petkov et al., 2006). Assuming that the human tonotopic axis is parallel to the HG long axis (Figure 1B), changes in stimulus frequency would be expected to have only minor effects on the geometric configuration of cortical AER sources, and therefore only minor effects on response characteristics. This is because sources would shift along the HG long axis—the direction of least morphological variation, perpendicular to local cortical curvature gradients.

More recent fMRI tonotopy studies, however (reviewed in Baumann et al., 2013; Besle et al., 2019; Saenz & Langers, 2014), have challenged the idea that the human tonotopic axis is oriented parallel to the HG long axis and thus rotated relative to that in macaques. These studies have consistently shown that in humans, like in macaques, regions of high- and low-frequency preference extend across the width of the STP (Figure 1C). Under the assumption that these regions represent tonotopic map endpoints—and thus tonotopic gradient reversals—this suggests that the human tonotopic axis runs across the width of HG, rather than along its length as suggested by the classical view. Direct evidence for this idea is provided by findings of our recent study combining ultra-high-field (UHF; 7 T) functional and microstructural MRI (Besle et al., 2019). We used tonotopic gradient reversals as *functional* markers of areal boundaries (analogous to using retinotopic gradient reversals for delineating visual cortical areas) and intra-cortical myelin content as an independent *structural* marker of the primary area, A1, receiving direct thalamic input. Both at single-subject and group levels, we found that A1 largely overlaps the functionally defined region bounded by the anterior-most high-frequency gradient reversal (labeled H_1_ in Figure 1C), located in the first transverse sulcus (just anterior to HG), and the posteriorly adjacent low-frequency reversal (L_1_), located on the posterior bank of HG, just behind the gyrus crest. This confirmed that, like in macaques, the human primary tonotopic axis is consistent with the predominant tonotopic axis across the wider auditory-responsive region, and that both axes run roughly perpendicular to the HG long axis rather than parallel to it. It also showed that tonotopic gradient reversals demarcate distinct architectonic areas and thus, similar to the visual domain (Warnking et al., 2002; Hinds et al., 2009), areas can be delineated functionally by locating tonotopic gradient reversals. The area behind A1, bounded by the low-frequency reversal on HG (L_1_) and the posteriorly adjacent second high-frequency reversal (H_2_) on planum temporale (PT), is less myelinated than A1 but exhibits other primary-like qualities such as sharp frequency tuning and may thus correspond to the histochemically defined “latero-posterior area” (LP; Wallace et al., 2002). As in A1, tonotopic gradients within LP run approximately perpendicular to the HG long axis, in a shallow V-shaped mirror-symmetric configuration with those in A1 (green arrows in Figure 1C). Irrespective of whether cortical AERs reflect simultaneous or separate contributions from these primary fields—or whether they also include non-primary contributions—these findings suggest that changes in stimulus frequency could potentially have substantial impact on underlying source geometry, as sources would be expected to shift in the direction of greatest morphological change (parallel to cortical curvature gradients). In area A1, for instance, increase in stimulus frequency should shift the activated cortical patch from near the HG crest, via the gyrus's anterior bank, toward the first transverse sulcus. Such shifts could dramatically alter both the orientation and overall strength of aggregate neuroelectric currents (Figure 1D), and thus the topography and amplitude of the resulting scalp responses.

This study aimed to utilize these new insights to explore whether cortical surface morphology can account for the well-known marked reduction in cortical AER amplitude with increasing stimulus frequency (reviewed in Tlumak et al., 2016). This reduction is a major feature of cortical AERs, which has critically influenced stimulus choices in previous studies (as a result, most cortical AER measurements have used lower stimulus frequencies—usually around 1 kHz; Näätänen & Picton, 1987) and sets cortical AERs sharply apart from subcortical AERs (auditory brainstem responses, or ABRs). Despite its obvious practical and potential theoretical importance, however, it remains without a satisfactory mechanistic explanation. To address this gap, the current study combines a rigorous meta-analytic characterization of the effect—including all known obligatory cortical AER components—with a novel forward-modeling approach informed by current knowledge of auditory cortical areal organization and tonotopic layout.

The meta-analysis combined an extensive semi-systematic literature search with a principled framework for both data preprocessing and statistical analysis based on linear mixed-effects (LME) modelling. The search included both early and late transient-evoked AER components, referred to as middle- and late-latency responses (MLR/LLR; Musiek & Nagle, 2018; Näätänen & Picton, 1987), the 40-Hz auditory steady-state response (ASSR; Picton et al., 2003), as well as selected subcortical AERs (ABR waves I, III, and V, and the 80-Hz ASSR; Jewett & Williston, 1971; Thornton & Coleman, 1975) for comparison. AER simulations combined a realistic EEG forward model based on the Colin27 stereotaxic average brain (Aubert-Broche et al., 2006) with computational simulations of tonotopic source distributions. The latter were derived from a functional parcellation of the human supratemporal auditory cortex and an idealized gridded representation of each area's tonotopic layout, based on high- (3 T) and ultra-high-field (7 T) blood oxygen level-dependent (BOLD) fMRI tonotopy data from 30 individual hemispheres. By integrating electrophysiological with fMRI tonotopic mapping data, the current study introduces a new approach to cortical AER source analysis, based on anatomically and functionally realistic source space constraints (see Cottereau et al., 2015; Hagler et al., 2009 for applications in inverse modeling), and generates new testable predictions for future experiments. The simulations provide a novel perspective of cortical AER generators and offer new insights into the mechanistic relationship between transient-evoked (MLR, LLR) and steady-state (40-Hz ASSR) cortical AERs.

## Methods

### Meta-Analysis

#### Literature Search

The frequency dependence of AERs has been narratively well established in the literature (e.g., Tlumak et al., 2016), and many relevant studies report this effect incidentally, embedded within studies with broader or different aims. For example, Naka et al. (1999) examined AERs across different sleep states and happened to include an awake condition with multiple stimulus frequencies. Thus, an exhaustive systematic review would have been disproportionately time-consuming and still unlikely to identify all relevant work. We therefore adopted a semi-systematic approach, in which one reviewer conducted the primary screening and a second reviewer verified the selections. As the extracted data were descriptive rather than inferential, tools for risk-of-bias assessment (e.g., funnel plots) were deemed unsuitable.

We started with a general literature search using the logical AND conjunction between the terms “frequency-specific” or “stimulus frequency,” “human” and one of several terms to denote auditory-evoked responses (“auditory-evoked potential/field/response” and “vertex potential”; see supplementary Table S1 for a full list of included search expressions and number of obtained records). Searches were performed on Google Scholar, PubMed, and the University of Nottingham's online library and allowed for any mention of the search terms anywhere in the article text. As several studies on frequency specificity date back to the early years of AER research, search date ranges were not restricted, allowing inclusion of any articles published in or before the year in which the search was conducted (2025). In addition, we also conducted separate informal searches for specific component labels, such as “middle-/late-latency response,” “N1P2,” or “auditory steady-state response.” Finally, we backward-searched relevant review articles, including Näätänen and Picton (1987) and Lütkenhöner et al. (2003b) for the LLR, Ross et al. (2003) and Picton et al. (2003) for the MLR and 40-Hz ASSR, as well as Tlumak et al. (2016) and Picton (2010) for all components generally.

The searches yielded 10,295 studies in total. Of these, 10,205 were rejected based on the relevance of their title, and a further 54 were rejected based on the relevance of their abstract or main text, yielding 36 studies that were ultimately included (see Figure S1 for a PRISMA diagram of the search procedure, and Table S2 for all included studies). Studies were included if they reported original (sensor-level) AER amplitudes for at least two different stimulus frequencies in either tabular or digitizable form. Two MEG studies (Ross et al., 2000; Ross et al., 2003) were included despite reporting source rather than sensor-level amplitudes, because the underlying source model was averaged across stimulus frequencies, and thus the possibility of confounding by frequency-related differences in source location (particularly depth) was minimized.

For each study, we extracted the group-average AER amplitudes for subjects with normal or reported-normal hearing and for supra-threshold stimulus intensity levels (i.e., levels that elicited clear, above-baseline responses). Graphical data were digitized using WebPlotDigitizer (Rohatgi, 2020). One study (Rothman, 1970) reported a mild hearing loss of 35 dB HL at 4 kHz in one subject, but the group-average data were nevertheless included because that subject's individual data followed the same trend as those of the normal-hearing subjects.

In EEG studies, LLR amplitudes were mostly measured as N1-P2 peak-to-peak amplitude difference, and MLR amplitudes as the difference between the Pa (the first MLR peak) and the subsequent Nb trough. Where relevant positive and negative peak amplitudes were reported separately, we combined them by calculating their positive difference (e.g., P2–N1). For MEG studies, we used the reported N1m and Pam peak RMS amplitudes (here, “m” stands for “magnetic”). Where MEG studies reported amplitudes separately for each hemisphere, amplitudes were averaged across hemispheres.

We also extracted, as accurately as possible, the number of subjects contributing to these averages (N in Table S2). Because many older studies did not consistently report experiment- or condition-specific sample sizes, these numbers should be regarded as approximate. When available, we used the sample size explicitly reported for the specific figure or result being digitized; otherwise, we used the reported overall study sample size. In cases where undeclared subsets of subjects were used for different experiments or conditions, the overall sample size will overestimate experiment- or condition-specific sample sizes.

#### Data Preprocessing

##### Correction for Variation in Hearing Level

Included studies differed in how stimulus intensities were matched across frequencies, with some studies using a constant hearing or sensation level (HL/SL; i.e., expressed relative to normal or individual auditory thresholds) and others using a constant absolute (or physical) level (sound pressure level, SPL). Because auditory thresholds vary across frequencies, HLs or SLs corresponding to constant SPLs will also vary, and this could bias the frequency dependence of respective AER amplitudes. To minimize such bias, data sets using constant SPLs were corrected for frequency-dependent HL variation. First, we converted SPLs to actual HLs using the monaural minimal audible pressure (MAP) values from Moore et al. (1997). As the MAP varies across frequencies, so did the derived actual HLs. Then, we fitted all log-transformed AER amplitudes (in their original units; referred to as *lgamp* in the model equation below) with a linear mixed-effects (LME) model, *lgamp* ∼ *lgfrq* + *lgfrq*^2^ + *hl* + (1|*study*), containing fixed effects of linear and quadratic frequency (also in logarithmic units; *lgfrq* and *lgfrq*^2^), a linear effect of actual HL (*hl*), and random by-study intercepts (1|*study*). The model was fitted for each AER component separately (ABR and 80-Hz ASSR, grouped collectively as ABR, MLR, LLR, and 40-Hz ASSR) using all available (constant-HL/SL and constant-SPL) data sets. For each constant-SPL data set, we then created two sets of predicted amplitudes, one for the actual HL values (derived from MAP), and another for a hypothetical constant HL, corresponding to the average actual HLs across frequencies. Finally, to create SPL-corrected amplitudes (i.e., corrected for HL variation across frequencies), we divided the actual reported amplitudes (in linear units) by the predicted amplitudes for the actual (varying) HLs and multiplied by the predicted amplitudes for the hypothetical (constant) HL.

For convenience, in this and the other preprocessing steps, LME models were implemented using Matlab R2025b (The MathWorks, Inc., Natick, MA, USA) via the *fitlme* function in the Statistics and Machine Learning Toolbox. The models were fitted using restricted maximum likelihood (REML) estimation and “effects” coding.

##### Conversion to Microvolt Units

Using the same model, we also converted all amplitudes not originally reported in μV to μV—the standard unit for EEG responses. In this case, the LME model was fitted using only amplitudes already in μV and then estimated using the parameters (stimulus frequencies and levels) of the non-μV amplitudes. Conversion to μV was then performed by dividing the non-μV amplitudes for each study by their geometric mean (across frequencies and levels) and multiplying by the geometric mean of the corresponding estimated amplitudes.

##### Correction for Frequency-Unrelated Effects

To visualize the frequency-dependence of AER amplitudes without irrelevant effects of stimulus HL and study, we corrected each data set—for each component, study, and stimulus HL—for any fixed or random effects of HL, as well as random effect of study. First, we fitted an extended LME model, *lgamp* ∼ *lgfrq* + *lgfrq*^2^ + *hl* + (1|*study*) + (*hl*−1|*study*) + (*lgfrq*−1|*study*) + (*lgfrq*^2^−1|*study*), also including random by-study slopes for all fixed effects (e.g., *hl*−1|*study*, etc.; here, “−1” signifies that random slopes and intercepts were uncorrelated) to all SPL-corrected and μV-transformed amplitudes for a given component. For each data set of each study, we then created two model predictions, both using 1.4142 kHz (the geometric mean between 0.25 and 8 kHz) as stimulus frequency: one conditional on study and using the data set's own HL value, and the other marginal and using a standardized HL value of 60 dB. Correction was then performed by dividing the original (SPL-corrected and μV-transformed) amplitudes by the conditional prediction and multiplying by the marginal one.

##### Standardization Across Components

To visualize differences in component-specific amplitude–frequency relationships across components, we normalized each data set for each component by the geometric mean of that component's average relationship across six octave-spaced frequencies between 0.25 and 8 kHz—the range most commonly represented across included studies. Note that, because amplitude–frequency relationships were represented in double-logarithmic units (*lgamp* and *lgfrq*), this normalization does not alter the relationship slopes in decibels per octave; it merely shifts the relationships vertically (along the ordinate), so relationships coincide at the geometric center of the 0.25–8 kHz frequency range (1.4142 kHz).

#### Statistical Analysis

Statistical comparisons between response components (ABR, MLR, LLR, and 40-Hz ASSR) and measurement modalities (EEG, MEG) were performed using extended versions of the LME models used for data preprocessing, allowing for two-way interactions between frequency (*lgfrq* and *lgfrq*^2^) and level (*hl*) on the one hand, and component (*comp*) or measurement modality (*modl*), where appropriate, on the other hand. The models were fitted to the original AER amplitudes (after SPL-correction and conversion to μV) and were implemented using the *lmer* function from the *lme4* and *lmerTest* packages in R (Bates et al., 2015; Kuznetsova et al., 2017; R Core Team, 2025). The quadratic frequency term (*lgfrq*^2^) was orthogonalized with respect to the linear term (*lgfrq*) using a Gram–Schmidt procedure to only capture variance not already explained by the linear term and eliminate collinearity between the terms.

LME models were first fitted to the data for each component separately, to test the significance of the linear and quadratic frequency trends. Significance was tested using likelihood ratio tests reported as *χ^2^* statistics with associated *P* values and Bayes factors (Table S3). The latter quantify the strength of evidence in favor of the alternative hypothesis relative to the null and thus provide an interpretable measure even when results are not statistically significant. Likelihood ratio test results were verified using semi-parameteric *wild* bootstrapping (5,000 resamples; *lmeresampler* package; Loy et al., 2023), which respects the nested covariance structure of the mixed-effects model while preserving the full parameter space (stimulus frequencies and levels) of the original data for each resample, thereby avoiding singular model fits (rank-deficient variance-covariance matrices) that would otherwise arise because different studies contributed data for different frequency and level ranges. The single-component models included the fixed main effects of linear and quadratic frequency (*lgfrq*, *lgfrq*^2^) and level (*hl*) as well as the by-study random intercepts and any random slopes that significantly improved the model fit. The latter were assessed using restricted-maximum-likelihood (REML) likelihood ratio tests via the *ranova* function which is part of the *lmerTest* package. For the ABR, random slopes were required for *hl*, *lgfrq* and *lgfrq*^2^; for the 40-Hz ASSR and LLR, random slopes were required for *hl* and *lgfrq*; and for the MLR, only the random intercept was necessary. For none of the components did a correlated random intercept–slope structure improve the model fit.

To test differences in the linear frequency–amplitude slope between the ABR on the one hand and the cortical components (MLR, LLR, and 40-Hz ASSR) on the other, we fitted the respective data with models that included the by-component interactions with *hl*, *lgfrq*, and *lgfrq*^2^ as fixed effects, together with the random-effects structure identified for the ABR: *lgamp* ∼ (*hl* *+* *lgfrq* + *lgfrq*^2^)*comp + (1|*study*) + (*hl*−1|*study*) + (*lgfrq*−1|*study*) + (*lgfrq*^2^−1|*study*). We then tested the linear frequency-by-component interaction (*lgfrq*:*comp*) using likelihood ratio tests and semi-parametric *wild* bootstrapping (5,000 resamples; Table S4).

To test differences in linear frequency–amplitude slope among cortical components (MLR, LLR, and 40-Hz ASSR), we fitted a similar model to the one described above, but excluding the random slope for *lgfrq*^2^, which was non-significant for the cortical components. This model was fitted both to the data for all three cortical components and, separately, to the data for the transient-evoked components (MLR and LLR) only (Table S5). The purpose of this restricted model fit was to determine whether the significant frequency-by-component interaction observed in the initial model was driven primarily by the 40-Hz ASSR.

Finally, to compare the frequency–amplitude dependence between EEG and MEG measurements, we fitted a model that included by-modality (EEG vs. MEG) interactions with *hl*, *lgfrq*, and *lgfrq*^2^ as fixed effects, together with by-study random intercepts and random slopes for *hl* and *lgfrq*: *lgamp* ∼ (*hl* *+* *lgfrq* + *lgfrq*^2^)*modl + (1|*study*) + (*hl*−1|*study*) + (*lgfrq*−1|*study*). This model was applied to the data for the LLR component, for which EEG and MEG studies were most evenly represented. To assess modality-related differences not only in the average (linear) frequency–amplitude slope but also the relationship shape, we tested the by-modality interactions with both the linear and quadratic frequency terms (Table S6).

In quantitative meta-analysis, individual studies are typically combined using precision-based weights, most commonly the inverse of each study's sampling variance. Because most studies did not report a measure of variance for their amplitude estimates, the number of study participants (N in Table S2) was used as a proxy for sampling precision. Under standard meta-analytic assumptions, larger samples yield lower sampling variance; thus, weighting the LMEs by study sample size approximates inverse-variance weighting and ensures that larger studies exert proportionally greater influence on the estimates. Supplementary Tables S3–S6 show that the weighted statistical results were consistent with the corresponding unweighted results, although generally more conservative (larger *P* values). The Results section reports the weighted results. For repeated tests (the separate model fits to each component and the pairwise comparisons between ABRs and cortical components), familywise type-I error rate was controlled using the Holm–Bonferroni method.

### MRI Data Acquisition and Analysis

#### Participants

Functional and structural MRI data were acquired from 15 normal-hearing participants (nine male; mean age = 28.99 ± 7.65 years, SD). All participants reported no history of audiological or neurological disorders and provided written informed consent prior to scanning. Imaging was performed on 3-T Philips Ingenia wide-bore (seven participants) and 7-T Philips Achieva (eight participants) scanners at the University of Nottingham's Sir Peter Mansfield Imaging Centre (SPMIC), both equipped with 32-channel receive coils. Experimental procedures were approved by the University of Nottingham Faculty of Medicine and Health Sciences Research Ethics Committee.

#### Image Acquisition

**Figure 2.**
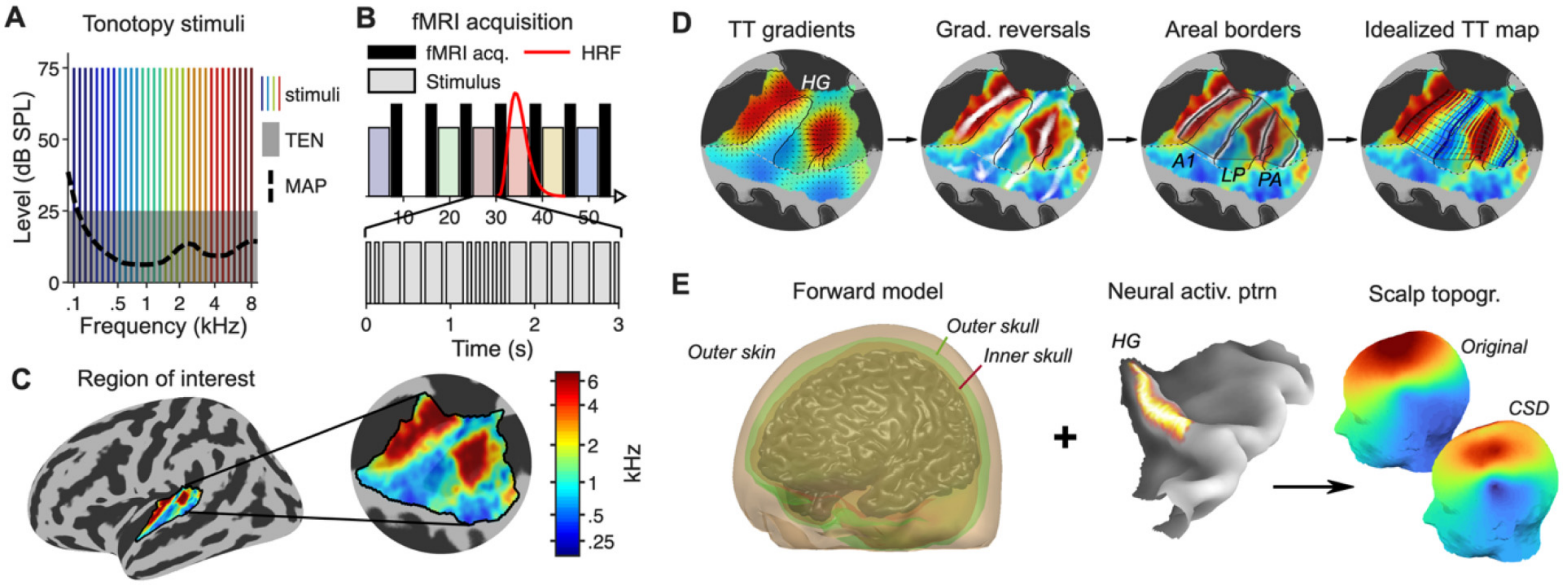
Tonotopic mapping (A–C), functional parcellation (D) and auditory-evoked response (AER) forward simulations (E). (A) Tonotopy stimuli (colored lines; 32 narrowband noises grouped into eight blocks of four for general linear modeling). Gray highlight: continuous threshold-equalizing noise (TEN) used to minimize sensation-level variation across stimuli; black dashed line: normal absolute (in-quiet) hearing threshold (“minimum audible pressure level,” MAP) in decibel sound pressure level (dB SPL). Ordinate: stimulus and TEN levels used at 3 T. (B) Pseudo-sparse imaging sequence; black bars: volume acquisitions; colored bars: stimuli; red line: hemodynamic response function (HRF) to scanner noise. The inset shows the Morse code-like pattern of stimulus bursts. (C) Auditory-responsive region of interest (ROI) with minimally smoothed (2.5 mm) probabilistic tonotopic map (color bar) on inflated (left) and flattened (right) *fsaverage* left-hemisphere cortical surfaces. (D) Functional parcellation into areas A1, LP, and PA: (a) computation of vertex-wise tonotopic (TT) gradients (arrows) using the 7.5-mm smoothed tonotopic map; (b) detection of gradient reversals (white highlight); (c) identification of largest connected subcomponents to define areal borders; and (d) construction of idealized tonotopic grids. (E) AER forward simulations: a realistic EEG forward model (left) was combined with simulated neural activation patterns (middle) to generate AER scalp topographies (right, top) and associated current source densities (CSDs; right, bottom). HG: Heschl's gyrus.

Functional images were acquired across two runs lasting about 9 min each. Images were acquired using a pseudo-sparse T2*-weighted single-shot gradient-echo (GE) echo-planar imaging (EPI) sequence. Each volume comprised 24 oblique-axial slices, oriented parallel to the Sylvian fissure, with 1.5 mm isotropic voxel resolution [TE: 40 ms (3 T) / 25 ms (7 T); flip angle: 90°; SENSE factor: 3; FOV: 156 × 36 × 174 mm^3^; phase-encoding direction: AP]. Image acquisition time (TA) was 2 (3 T) or 1.5 s (7 T), and volumes were spaced by a 7.5 s repetition time (TR), allowing sound stimuli to be presented during the silent intervals in between volumes (Figure 2B). To minimize adaptation, stimuli were presented in a Morse code-like pattern of bursts (random 50- or 200-ms bursts separated by 50-ms silent periods; Thomas et al., 2015; Figure 2B, inset). Stimuli were narrowband noises centered at 32 different frequencies between 0.1 and 8 kHz, spaced quasi-logarithmically according to the human cochlear frequency-position function based on simultaneous notched-noise masking (the so-called ERB-number, or NERB, scale; Moore, 1986). Stimulus bandwidths matched the cochlear filter width at the stimulus center frequency, expressed as equivalent rectangular bandwidth (ERB; Glasberg & Moore, 1990). Stimuli were presented at 75 (3 T) or 80 dB SPL (7 T) and accompanied by continuous 25- (3 T) or 35-dB SPL (7 T) threshold-equalizing noise (TEN; Moore et al., 2004) to maintain a constant sensation level of 50 (3 T) or 45 dB (7 T) across frequencies (Figure 2A). At the lowest frequencies, effective sensation levels were likely lower, particularly at 3 T, as the normal absolute (in-quiet) threshold level (bold dashed line in Figure 2A) exceeded the 25/35-dB SPL threshold level induced by the TEN.

T1-weighted structural images were acquired using either an MPRAGE sequence (at 3 T; TE: 3.8 ms; TR: 8.2 ms; flip angle: 8°; FOV: 256 × 162 × 256 mm^3^) or a phase-sensitive inversion recovery (PSIR) sequence (at 7 T; TE: 3.76 ms; TR: 13.0 ms; TI1/TI2: 791/2391 ms; flip angle: 8°; FOV: 192 × 140 × 184 mm^3^). Image resolution was 1 mm isotropic at 3 T, and 0.8 mm at 7 T, except for one participant, where a resolution of 1 mm was used. PSIR images were downsampled to 1-mm resolution and used to generate semi-quantitative maps of longitudinal relaxation rate, R1 (van de Moortele et al., 2009), which, in turn, was used to measure intra-cortical myelin content (Shams et al., 2019).

#### Image Preprocessing and Analysis

Functional image time series were preprocessed by (a) thermal noise suppression using NORDIC (Vizioli et al., 2021), (b) head-motion correction with FSL's (Jenkinson et al., 2012) *mcflirt*, (c) correction for dynamic B_0_ inhomogeneity-related distortion using an in-house Matlab implementation of the methods described by Hahn et al. (2009), Kadah and Hu (1997) and Lamberton et al. (2007), and (d) temporal high-pass filtering at a 100-s cutoff with FSL's *fslmaths* to remove low-frequency drift. Stimulus-related response sizes were estimated at the single-subject level using a general linear model (GLM) implemented with FSL's *film_glm*, and converted to percent signal change. Each GLM contained eight regressors, with each regressor representing four consecutive stimulus frequencies from the original set of 32 (Figure 2A). The center frequencies of the eight sets ranged from 0.16 to 6.8 kHz.

Functional responses and semi-quantitative R1 values were projected to individual cortical surfaces, reconstructed using FreeSurfer (v8.0.0; Fischl, 2012). Individual R1 maps were corrected for linear effects of individual cortical curvature and thickness (Besle et al., 2019). Finally, functional and R1 values were projected from the individual surfaces to the left hemisphere of FreeSurfer's symmetrical *fsaverage* surface using individual cortical curvature-based spherical registrations (*mris_apply_reg*). Functional responses were averaged across the entire depth of the cortical gray-matter ribbon (between white-matter and pial surfaces), and R1 values were averaged over the ribbon's central half thickness.

#### Construction of Probabilistic Tonotopic map

Both functional and structural (R1) data were evaluated within an auditory-responsive region of interest (ROI), defined by taking the semi-convex hull of all vertices showing significant activation to any stimulus frequency (F-test; *P* ≤ .001, uncorrected) in at least half of all (30) individual hemispheres (Figure 2C). A tonotopic map was defined for each individual hemisphere by assigning each ROI vertex the stimulus frequency eliciting the maximum response (“preferred frequency”). Individual maps were then combined across hemispheres using a maximum-probability criterion (i.e., by assigning each vertex the frequency preferred in the majority of hemispheres; O'Neill et al., 2020), and the resulting probabilistic tonotopic map was spatially smoothed using 2.5- and 7.5-mm (full width at half maximum, FWHM) surface-based (2D) Gaussian smoothing kernels (*mri_surf2surf*). A circular patch was defined around the ROI's Jordan center (the graph-theoretical equivalent of the centroid), fully encompassing the ROI, and projected onto a planar (2D) circle using Tutte's algorithm (Tutte, 1963; Figure 2C, right panel), as implemented in Matlab by Muir (2025).

#### Functional Parcellation

Following similar methodology as employed in the visual domain (Wandell & Winawer, 2011), we used reversals in tonotopic gradient direction to delineate functionally defined tonotopic subdivisions of the supratemporal auditory region (the ROI defined in the previous section). For that, vertex-wise tonotopic gradients were derived from the 7.5-mm smoothed and flattened version of the probabilistic tonotopic map (Figure 2D, leftmost panel) using a procedure proposed by Mancinelli et al. (2019). The procedure involves first calculating face-wise gradients using the “per-cell linear estimation” (PCE) method and then averaging these across each vertex's immediate set of connected faces (“first-order ring”). Using the same procedure, gradients were also calculated for individual tonotopic maps.

Gradient reversals were located using an automated procedure similar to that proposed by Schönwiesner et al. (2015). In essence, the procedure calculates the likelihood of a given vertex to represent a gradient reversal by counting the proportion of directions—between 0° and 180° in 1° increments—along which the vertex exhibits an inversion in gradient direction (reversal in gradient sign) with respect to neighboring vertices. The resulting reversal likelihoods were thresholded at 11% (reversals in at least 20 of the 180 possible directions) to isolate the most likely reversal locations (Figure 2D, second-from-left panel). Using graph-theoretical methods (Matlab's *conncomp*), above-threshold vertices were then grouped into connected sets, and the four largest sets were taken to represent areal boundaries (Figure 2D, second-from-right panel). Each boundary was replaced by a sharp border using piecewise linear regression. The borders delineated three areas, labeled A1, LP, and PA, wholly comprised within the STP, and each containing a distinct and complete tonotopic map.

#### Derivation of Population Receptive Fields

Vertex-wise frequency tuning curves, or “population receptive fields” (pRFs; Dumoulin & Wandell, 2008) were derived using a non-parametric split-half cross-validation procedure based on responses from each of the two acquired functional runs. The procedure assigned each vertex a run-specific preferred frequency and then recentered responses from the other run with respect to that frequency. The recentered responses were averaged across runs and vertices within each area, and the resulting area-average pRFs were fitted with a mixture of two rounded-exponential (roex) functions sharing the same mode (to capture the pRFs’ sharper tip and broader tail). The functions were defined on the NERB scale (the frequency scale of the stimulus center frequencies). The fitted functions were used to derive the pRFs’ widths (FWHM) and expressed as a fraction of the audible frequency range (from 20 Hz to 20 kHz).

#### Statistical Analysis

Area-related differences in average vertex-wise frequency tuning width and semi-quantitative R1 values were assessed by fitting LME models with the *lme4* and *lmerTest* packages for *R*. For the R1 values, the model included the main effects of cortical area (A1, LP, and PA) and hemisphere (left and right), both coded as “effects,” as well as a random intercept for subjects: *R1* ∼ area + hemi + (1|*subj*). Type-III Wald F-tests with Kenward-Rogers-adjusted degrees of freedom indicated that hemisphere provided at least a marginal improvement in model fit. The area-by-hemisphere interaction, however, did not significantly improve the fit and was therefore not included. Random slopes for area or hemisphere likewise did not improve model fit (assessed using REML likelihood ratio tests) and were thus also excluded.

For the pRF widths (*FWHM*), the model included only area as a fixed effect, because neither hemisphere nor B_0_ field strength, nor their interactions with area, contributed significantly to the model fit. As for R1, the model for FWHM included a random intercept for subjects: *FWHM* ∼ *area* + (1|*subj*).

The effects of area on R1 and pRF width were tested using likelihood ratio tests and verified using semi-parametric *wild* bootstrapping (5,000 resamples). Pairwise differences between areas (A1-LP, A1-PA, LP-PA) were tested with paired t-tests. Familywise error rate was controlled using Holm-Bonferroni correction (Table S7).

### Forward Simulations

Forward simulations of AER amplitudes and scalp topographies were generated by combining an *EEG forward model*—comprising a realistic head volume conductor and source model—with simulated frequency-specific neural activation patterns within each functionally parcellated area (A1, LP, PA) or subsets of these areas. Given the sparseness of available MEG studies, particularly for the MLR and 40-Hz ASSR, as well as the disparities in MEG sensor types (gradiometers vs magnetometers) and sensor layouts (linear, circular or whole-head) across studies (see *Results: Meta-Analysis*), we did not explicitly simulate MEG responses. Instead, we used the current source densities (CSDs; Kayser & Tenke, 2015) of the simulated EEG responses to obtain a reference-free estimate of underlying source strength, independent of sensor arrangement. Frequency-specific neural activation patterns were derived from *idealized representations* of each region's tonotopic layout.

#### EEG Forward Model

To create a forward model for cortical AERs, we used the 2008 high-resolution version (Aubert-Broche et al., 2006) of the Colin27 stereotaxic average brain (Holmes et al., 1998)—an individual brain nonlinearly aligned to match the MNI305 stereotaxic template—to create a head volume conductor model and cortical source model. The head model was generated using the boundary element method, by constructing triangulated surface meshes representing boundaries between tissues of different electrical conductivities (brain, skull, and skin; Figure 2E, left panel). The inner and outer skull, as well as outer skin boundaries, each consisting of 2652 vertices, were generated using FSL's *betsurf* and converted from FSL to FreeSurfer coordinate space using Matlab. To generate the brain boundary, we reconstructed the Colin27 brain's smooth-white and pial cortical surfaces using FreeSurfer and, from these, created the mid-cortical surface as the mid-point along local surface normals. MNE-Python (v1.10.2; Gramfort et al., 2013) was used to synthesize the volume conductor model and generate the source model, using the standard MNE conductivities for each tissue type (0.3, 0.006, and 0.3 for brain, skull, and skin, respectively). The source space was defined by the vertices of the mid-cortical surface, with source orientations constrained to be perpendicular to the local cortical surface tangent (i.e., parallel to the surface normals). For inverse modeling, source density is typically reduced through downsampling to make associated matrix operations more manageable (e.g., Baillet, 2022). Here, no downsampling was applied to ensure adequate source density within the auditory-responsive supratemporal region (within this region, Euclidean distances between neighboring vertices averaged 0.9 mm). The leadfield matrix, *L*, was calculated for 36 electrodes from the extended EasyCap-M1 74-channel montage (based on the international 10–5 system: Fp1, Fp2, AFz, F7, F3, Fz, F4, F8, FT9, FC5, FC1, FCz, FC2, FC6, FT10, T7, C3, Cz, C4, T8, TP9, CP5, CP1, CP2, CP6, TP10, P7, P3, Pz, P4, P8, PO9, O1, O2, PO10, Iz).

#### Construction of Idealized Tonotopic Maps

To simulate activation patterns for arbitrary stimulus frequencies (i.e., frequencies not contained within the original fMRI tonotopy data), we created idealized, finely gridded representations of each area's tonotopic layout on the flattened cortical patch and then used these representations to infer vertex-wise preferred frequencies. First, we divided each border representing a gradient reversal into N = 100 equal-length segments. Based on the assumption that cortical tonotopic maps represent the entire audible frequency range, low- and high-frequency gradient reversals (shown in dark red and blue color in Figure 2D, rightmost panel) were assigned preferred frequencies of 20 Hz and 20 kHz, respectively. To construct the intervening tonotopic gradients (or preferred-frequency progressions), we assumed that progressions follow geodesic (shortest) paths between corresponding segments in neighboring borders (Figure 2D, rightmost panel). Like the borders, geodesics were divided into M = 100 equal-length segments, and each segment was assumed to represent equally distinguishable frequency differences, Δf (see Greenwood, 1990, and Daniel & Whitteridge, 1961, for the origin of this assumption in the context of the cochlear “frequency-position function” and the visual “cortical magnification factor,” respectively). Based on this assumption, segments were linearly mapped to the antiderivative (integral) of the reciprocal of Δf , $g(f)=\int_{0}^{f}\;1/\Delta f\;df$ . Here, 1/Δf represents the cortical magnification factor for frequency (i.e., the cortical distance corresponding to a unit frequency difference) and *g*(*f*) represents the cortical frequency-position function.

In the first instance, Δf was equated to the equivalent rectangular auditory filter bandwidth (ERB) as defined by notched-noise masking, Δf=24.67⋅(4.37⋅f+1)/1000 (Glasberg & Moore, 1990)—effectively assuming that cortical frequency magnification is linearly related to cochlear frequency magnification. In this case, g(f) equals the NERB scale, $g(f)=\frac{1000\cdot \ln(10)}{24.67\cdot 4.37}\cdot \log10(4.37\cdot f+1)$ , where “ln” is the natural logarithm. In two further simulations, Δf was equated to (a) a more recent estimate of cochlear frequency resolution based on otoacoustic emissions latencies, $\Delta f=f^{0.7}/12.7$ (Shera et al., 2002), yielding $g(f)=f^{0.3}/(12.7\cdot 0.3)$ , and (b) the frequency discrimination threshold, or “frequency difference limen” (DLF), which, at a sensation level of 50 dB and a stimulus duration of 1000 ms, can be characterized as $\Delta f=10^{0.38\cdot f^{0.82}-3.1422}$ (Micheyl et al., 2012), the reciprocal of which was integrated numerically using Matlab's integral function.

For each area, the difference between the average orientation of the geodesics and the mean of the individual area-average tonotopic gradient directions was tested with a circular one-sample t-test based on a large-sample normal approximation of the sample mean direction, implemented using the *circ_confmean* function of the CircStat circular statistics toolbox for Matlab (v2012a; Berens, 2009). The resulting *P* values were Holm-Bonferroni-corrected for familywise error rate across areas.

#### Simulation of Neural Activation Patterns

Neural activation patterns were simulated for six octave-spaced frequencies between 0.25 and 8 kHz and for each functional area (A1, LP, and PA) separately. To generate an activation pattern for a given frequency and area, we assigned each grid point, *i* and *j*, i∈[0,N] and j ∈[0,M] , in the area's idealized tonotopic map a source strength, defined by the Gaussian function value, $\exp(-{\left(g(\;f_{i,j})-g(\;f_{Stim})\right)}^{2}/(2\cdot \sigma^{2}))$ , of the difference between the cortical distances, $g(\;f_{i,j})$ and $g(\;f_{Stim})$ , corresponding to the point's assigned preferred frequency value, $f_{i,j}$ , and the stimulus frequency, $f_{Stim}$ . The spread of the Gaussian ( σ ) was constant across frequencies and was either varied parametrically or based on the estimated width (FWHM) of the respective area-average pRF [ $\sigma =\mathrm{FWHM}/\left(2\cdot \sqrt{2\cdot \ln(2)}\right)]$ . Activation patterns were interpolated to the flat-patch vertices using nearest neighbors, and, from there, projected to the forward-model source space (left and right hemispheres of the Colin27 mid-cortical surface).

Because activation patterns were defined with the same peak amplitude (i.e., 1), and the same spatial width, across frequencies, and because vertices within the cortical mesh are approximately equidistant, all frequencies produced the same current density (i.e., summed current strength per unit cortical area). At the tonotopic map boundaries, activation patterns could be partially truncated; to avoid artificially reducing their total strength, we scaled each pattern by the inverse of its truncation fraction.

#### Transversal Subdivision

**Figure 3.**
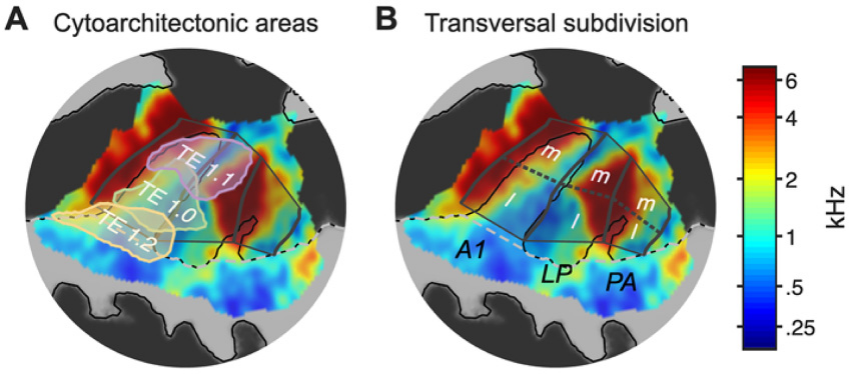
Transversal subdivision of auditory cortical areas suggested by post-mortem cytoarchitectonic data. (A) Cytoarchitectonic subdivisions of human auditory cortex (TE1.0, TE1.1, TE1.2) as defined by Morosan et al.  (2001) overlaid on the current probabilistic tonotopic map. Each TE field is visualized using its probabilistic map, as implemented in FSL, thresholded at an area-specific quantile, chosen such that all fields are displayed with equal surface extent despite differences in their probabilistic variability. (B) Simplified transversal subdivision of areas A1, LP, and PA into medial (m) and lateral (l) halves used in the current simulations.

Post-mortem cytoarchitectonic data (Morosan et al., 2001) have suggested that the primary tonotopic gradients in human auditory cortex may not represent single, unitary fields but could instead be subdivided transversally, parallel to the tonotopic axis (Figure 3A). The borders of such transversal subdivisions would run approximately parallel to the tonotopic axis and would therefore remain undetected when defining areas solely on the basis of tonotopic gradient reversals. To explore the potential impact of such a subdivision on our analyses, we carried out a simple proof-of-principle test in which the reversal-defined areas A1, LP, and PA were each split into medial and lateral halves (Figure 3B).

#### Derivation of Simulated Response Amplitudes

Neural activation patterns were combined with the leadfield matrix, *L*, to create scalp voltage distributions (scalp topographies; Figure 2E right panel, top). EEG response amplitudes were simulated by taking the difference between the vertex (Cz) and average mastoid (TP9 and TP10) channels (equivalent to “linked mastoids”). Supplementary Figure S2 demonstrates that the predicted frequency dependence is largely insensitive to common referencing schemes, including linked mastoids and average reference. To create a reference-free estimate of the net strength of responses’ underlying neuroelectric currents, we also calculated the root-mean-square amplitude of the scalp voltages’ second spatial derivative, or surface-Laplacian, referred to as current source density (CSD). CSDs were generated using the spherical spline method implemented in the CSD Toolbox (Kayser, 2009; Kayser & Tenke, 2006). CSDs of scalp EEG responses reflect the size and direction of radial current flow at the scalp surface. For tangential sources, like those within supratemporal auditory cortex, CSD-transformed EEG data closely resembles MEG data in terms of topographic sharpness and localization (Nunez & Srinivasan, 2006; Pascual-Marqui et al., 1994). Linear fitting between observed and simulated amplitude–frequency relationships was performed using Matlab's constrained linear least-squares solver (*lsqlin*) with the default interior-point optimization algorithm. Graphical representations of scalp voltage and CSD distributions were generated using the *headplot* function in EEGLAB (v2025.0.0; Delorme & Makeig, 2004).

## Results

### Meta-Analysis

We identified 36 eligible studies. Most studies (21) reported LLR amplitudes, five studies reported MLR amplitudes, eight reported 40-Hz ASSR amplitudes, and four reported subcortical AER (ABR) amplitudes, which were included for comparison with the cortical responses. Two studies reported amplitudes for more than one component (Pantev et al., 1995: MLR and LLR; Tlumak et al., 2016: 40-Hz ASSR and LLR). Among the 36 studies, 24 used EEG, 13 used MEG and one (Tuomisto et al., 1983) used both modalities. Most MEG studies (10) reported LLR amplitudes, one reported MLR amplitudes, and three reported 40-Hz ASSR amplitudes. None reported frequency-specific ABR amplitudes. With few exceptions, EEG amplitudes were measured using the vertical montage (vertex-mastoid difference), and MEG amplitudes were measured as root-mean-square (RMS) amplitudes across sensors. Most MEG studies used first-order axial gradiometers arranged in circular arrays, although magnetometers and linear or whole-head array configurations were also used (see supplementary Table S2).

**Figure 4.**
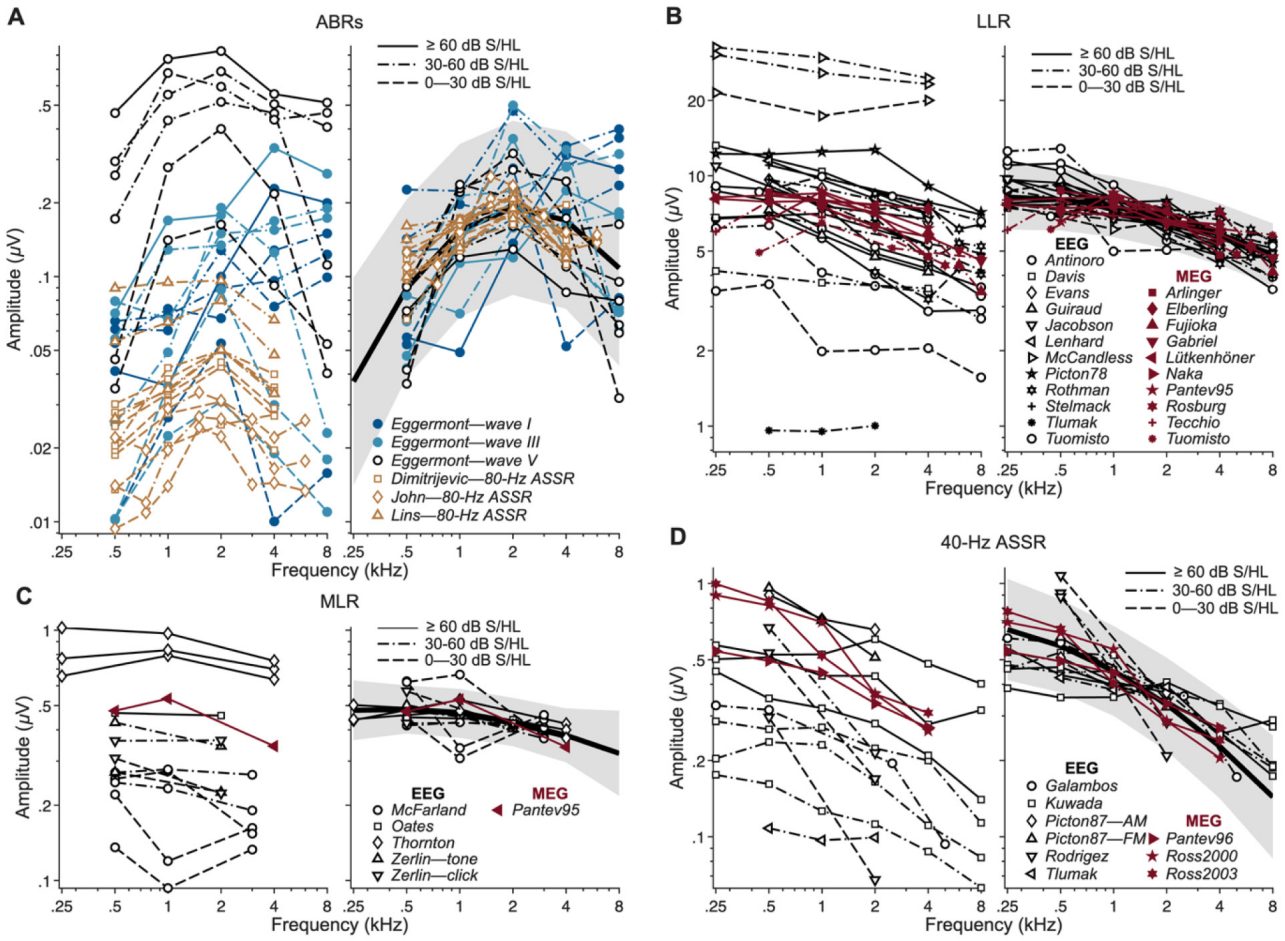
Meta-analytic data for each of four AER components. (A) Auditory brainstem responses (ABRs), including transient-evoked waves I, III, and V and 80-Hz auditory steady-state response (ASSR). (B) Late cortical AERs (late-latency response, LLR). (C) Early cortical AERs (middle-latency response, MLR). (D) 40-Hz ASSR. Symbols indicate different studies (bottom legends); line styles indicate stimulus intensity ranges in sensation or hearing level (S/HL; top legends). For ABRs, colors distinguish response types (waves I–V, 80-Hz ASSR); for cortical AERs (LLR, MLR, 40-Hz ASSR), colors distinguish EEG (black) versus MEG (red) data. Left panels: original digitized response amplitudes after SPL-correction and conversion to μV. Right panels: same data but corrected for frequency-unrelated effects of stimulus level and study. Bold black lines: average amplitude–frequency relationship for each component, derived from a linear mixed-effects (LME) model analysis; gray shading: parametric 95% CIs. *Antinoro*: Antinoro et al. (1969); *Arlinger*: Arlinger et al. (1982); *Davis*: Davis et al. (1968); *Dimitrijevic*: Dimitrijevic et al. (2002); *Eggermont*: Eggermont and Don (1980); *Elberling*: Elberling et al. (1982); *Evans*: Evans and Deatherage (1969); *Fujioka*: Fujioka et al. (2002); *Gabriel*: Gabriel et al. (2004); *Galambos*: Galambos et al. (1981); *Guiraud*: Guiraud et al. (2007); *Jacobson*: Jacobson et al. (1992); *John*: John et al. (2001); *Kuwada*: Kuwada et al. (1986); *Lenhard*: Lenhardt (1971); *Lins*: Lins et al. (1996); *Lütkenhöner*: Lütkenhöner et al. (2003b); *McCandless*: McCandless and Best (1966); *McFarland*: McFarland et al. (1977); *Naka*: Naka et al. (1999); *Oates*: Oates and Stapells (1997); *Pantev95*: Pantev et al. (1995); *Pantev96*: Pantev et al. (1996); *Picton78*: Picton et al. (1978); *Picton87*: Picton et al. (1987); *Rodrigez*: Rodriguez et al. (1986); *Rosburg*: Rosburg et al. (1998); *Ross2000*: Ross et al. (2000); *Ross2003*: Ross et al. (2003); *Rothman*: Rothman (1970); *Stelmack*: Stelmack et al. (1977); *Tecchio*: Tecchio et al. (2000); *Thornton*: Thornton et al. (1977); *Tlumak*: Tlumak et al. (2016); *Tuomisto*: Tuomisto et al. (1983); *Zerlin*: Zerlin et al. (1973).

Both cortical and subcortical AER amplitudes varied substantially across studies and across data sets within studies (with each data set corresponding to a fixed stimulus level; Figure 4, left panels). Much of this variability reflected effects of stimulus level and study-specific factors. Once these effects were accounted for (see *Methods: Meta-Analysis—Data Preprocessing*), the frequency dependence of AER amplitudes became remarkably consistent within components when both frequency and amplitude were expressed in logarithmic units (Figure 4, right panels).

**Figure 5.**
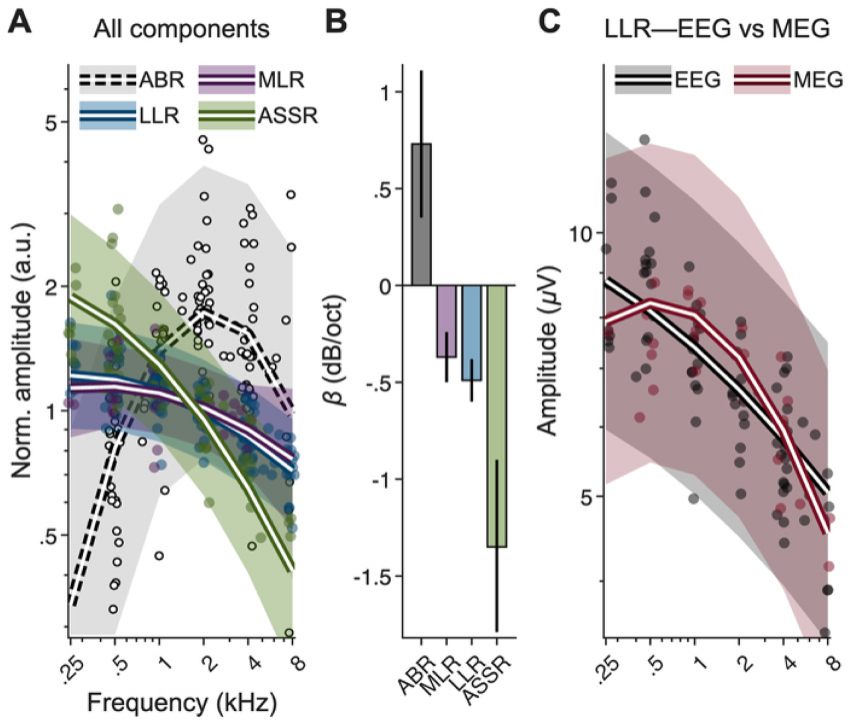
Comparison of amplitude–frequency relationships across components (A and B) and between EEG and MEG data (for the LLR; C). In panels A and C, bold, double-stroke lines with shaded areas show average amplitude–frequency relationships with parametric 95% CIs. Panel A shows the same relationships as Figure 4 but standardized across components (normalized by the geometric mean of each component's average relationship across frequencies; see *Methods: Meta-Analysis—Standadization Across Components*), whilst panel C shows new, modality-specific relationships. Data points in A and C are the corrected values from Figure 4 (right panels), jittered along the abscissa for clarity. Panel B shows the average linear slope (in decibels per octave) of the relationships shown in A with *wild*-bootstrap 95% CIs (error bars).

Characterization of the amplitude–frequency dependence of the MLR was limited by the small number of available studies, the few data sets within those studies, and the relatively small frequency range they covered. Nevertheless, the cortical AER data indicated a significant difference in the rate of frequency-related amplitude decline between transient-evoked components (MLR, LLR) and the 40-Hz ASSR (Figure 5A; component-by-frequency interaction: $\chi^{2}$ (2) = 18.09, *P* < .001, *BF* = 34.5), with the transient-evoked components showing a shallower rate (−0.35/−0.50 dB/oct) than the 40-Hz ASSR (−1.37 dB/oct; Tables S3 and S5), but no significant difference between each other [component-by-frequency interaction for MLR vs. LLR only: $\chi^{2}$ (1) = 1.80, *P* = .180, *BF* = 1/5.41].

Frequency-related amplitude reduction (significant negative linear association between log-transformed amplitude and frequency) was consistently observed across all cortical AER components (MLR, LLR and 40-Hz ASSR), but not ABRs (Figures 4 and 5A; Table S3). This difference was statistically supported by significant component-by-frequency interactions in all pairwise comparisons between ABRs and cortical components (*P* ≤ .002, Holm-Bonferroni-corrected; Table S4). ABR amplitudes showed a predominantly non-monotonic (inverted U-shaped) frequency dependence with a broad peak near 2 kHz, particularly for wave V and the 80-Hz ASSR and, at lower stimulus levels, also for waves I and III. At higher levels, wave-I and -III amplitudes increase with increasing frequency—opposite to cortical AER amplitudes (Eggermont & Don, 1980). For cortical components, frequency-related amplitude reduction tended to accelerate toward higher frequencies, supported by significant quadratic frequency effects for the 40-Hz ASSR and LLR (*P* ≤ .022; Table S3). Some MLR and LLR data sets showed a non-monotonic amplitude–frequency dependence, somewhat similar to ABRs, though less pronounced and peaking at a lower frequency (∼1 kHz).

MEG studies generally showed a similar amplitude–frequency dependence to EEG studies (compare red and black lines and symbols in Figure 4B–4D). This was most evident for the LLR, where the numbers of available EEG and MEG studies were the most comparable (12 and 10, respectively; Figure 5C). EEG and MEG LLR amplitudes did not differ significantly in the linear frequency effect [modality-by-frequency interaction: $\chi^{2}$ (1) = 0.06, *P* = .812, *BF* = 1/11.5]. However, MEG LLR amplitudes exhibited a more strongly accelerating amplitude–frequency relationship, as indicated by a significant interaction between modality and the quadratic frequency effect [ $\chi^{2}$ (1) = 4.61, *P* = .032]; Bayesian evidence for this interaction, however, was weak (*BF* = 1/1.18).

Taken together, the meta-analytic results indicate that frequency-related amplitude decline is a distinctive feature of cortical AERs, absent in subcortical responses. The shape—though not necessarily the rate—of this decline may differ between EEG and MEG responses, but evidence is limited. Unexpectedly, we also found differences in the rate of decline between transient-evoked and steady-state components, suggesting possible functional distinctions between these two response types. In the following sections, we use AER forward modeling to assess whether, or to what degree, these findings can be attributed to tonotopic organization across supratemporal cortical morphology.

### Functional Parcellation and Idealized Tonotopic Layouts

**Figure 6.**
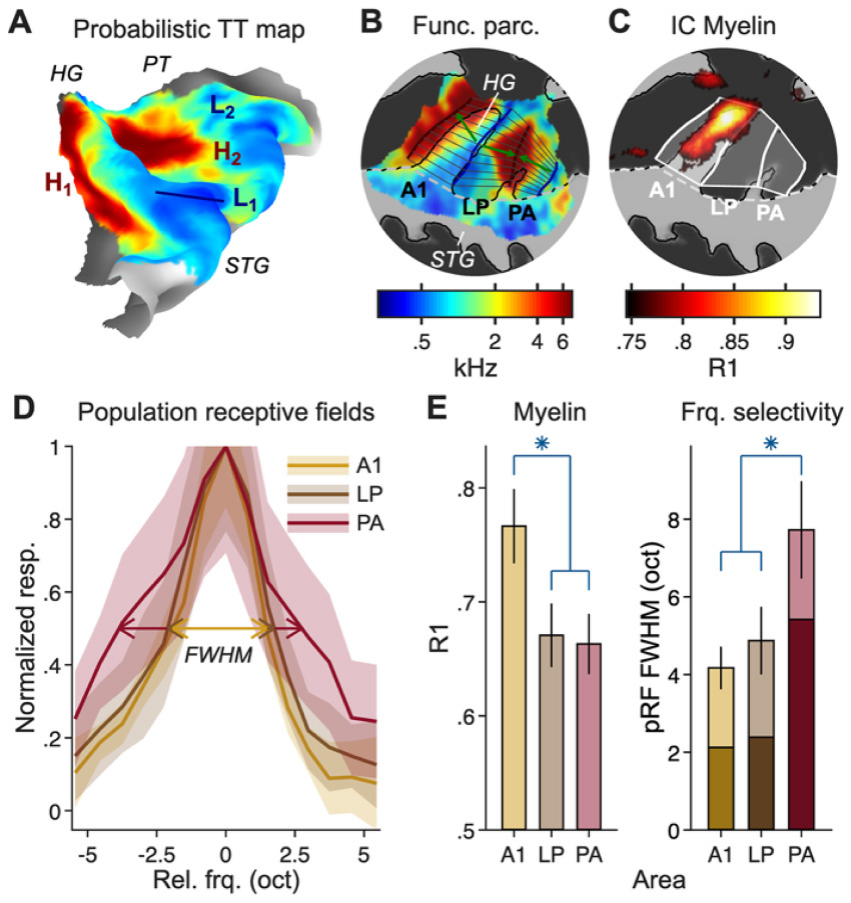
Probabilistic tonotopy, functional parcellation, and population receptive fields (pRFs). (A) Probabilistic tonotopic (TT) map across 30 individual hemispheres projected onto the left superior temporal gyrus (STG) of the Colin27 cortical surface. Low- and high-frequency regions (L_1/2_, H_1/2_) are highlighted in blue and red (color bar in B). (B) Tonotopic map and derived functional parcellation on the flattened *fsaverage* cortical patch. Dark red and blue lines: areal borders representing tonotopic gradient reversals; green arrows: area-average gradient directions; thin dark-gray lines: example geodesic paths for idealized tonotopy models. The light-gray dashed line shows the curvature inflection marking the STG boundary. (C) Average intra-cortical (IC) myelin (semi-quantitative R1 values, thresholded at the 90th percentile). (D) Area-average frequency pRFs with 95% CIs; arrows indicate the pRF's full width at half maximum (FWHM). (E) Area-average myelin (left) and pRF widths (right); error bars show 95% CIs. Darker-shaded bars (right panel) show estimated neural tuning widths.

Consistent with multiple previous fMRI tonotopy studies (e.g., Da Costa et al., 2011; Langers & van Dijk, 2012; Striem-Amit et al., 2011; Talavage et al., 2004), the probabilistic tonotopic map derived from the current 3 T and 7 T data showed two prominent high-frequency regions: H_1_, located in the first transverse sulcus, just anterior to HG, and H_2_, located on PT, just behind Heschl's sulcus (Figure 6A). Both regions spanned the width of the STP from its medial border with the circular sulcus of the insula to its lateral edge marked by the curvature inflection that delineates the superior temporal gyrus (STG). The high-frequency regions were separated by a large low-frequency region, L_1_, extending along the entire length of HG and further onto STG, and were posteriorly flanked by another, smaller low-frequency region, L_2_, on the posterior part of PT. The pattern suggested three functional areas (Figure 6B), henceforth referred to as A1, LP, and PA (“posterior area”), each with a distinct, complete tonotopic map (see *Methods: MRI Data Acquisition and Analysis—Functional Parcellation*). Consistent with previous findings, average tonotopic gradients within all three areas were oriented approximately perpendicular to the long axis of HG.

In line with the histochemical data of Wallace et al. (2002), the anterior-most area, A1, was significantly more highly myelinated than the other two areas [Figure 6C and E; main effect of area: $\chi^{2}$ (2) = 42.79, *P* < .001, *BF* > 150; A1 vs. LP/PA: *t*(15) ≥ 7.45, *P* < .001; LP vs. PA: *t*(15) = 0.55, *P* = .593; all *P* values Holm-Bonferroni-corrected; Table S7A], suggesting that this area receives highly myelinated thalamic afferents to its granular layer (IV), and is thus homologous to the macaque A1 area (Rouiller & Durif, 2004). Consistent with recent findings by Besle et al. (2019), both A1 and the posteriorly neighboring area, LP, occupying Heschl's sulcus, showed higher frequency selectivity (narrower frequency tuning width; Figure 6D and 6E) than the posterior-most area, PA [main effect of area: $\chi^{2}$ (2) = 36.10, *P* < .001, *BF* > 150; A1 vs. LP: t(25) = –2.02, *P* = .055; A1/LP vs. PA: t(23/24) ≤ −4.27, *P* < .001; Table S7B], suggesting that LP is part of the human auditory core (or primary) region, and thus likely represents the functional equivalent of the histochemically defined LP area (see Wallace et al., 2002).

To simulate neural activation patterns for frequencies not included in the fMRI tonotopy data, we constructed idealized, finely gridded representations of each area's tonotopic layout (see *Methods: Forward Simulations—Construction of Idealized Tonotopic Maps*). The idealized maps were based on the assumptions that (a) tonotopic frequency progressions run along geodesic (shortest) paths between corresponding tonotopic endpoints (gradient reversals; Figure 6B) and that (b) progressions represent the entire audible frequency range from 20 Hz to 20 kHz.

**Figure 7.**
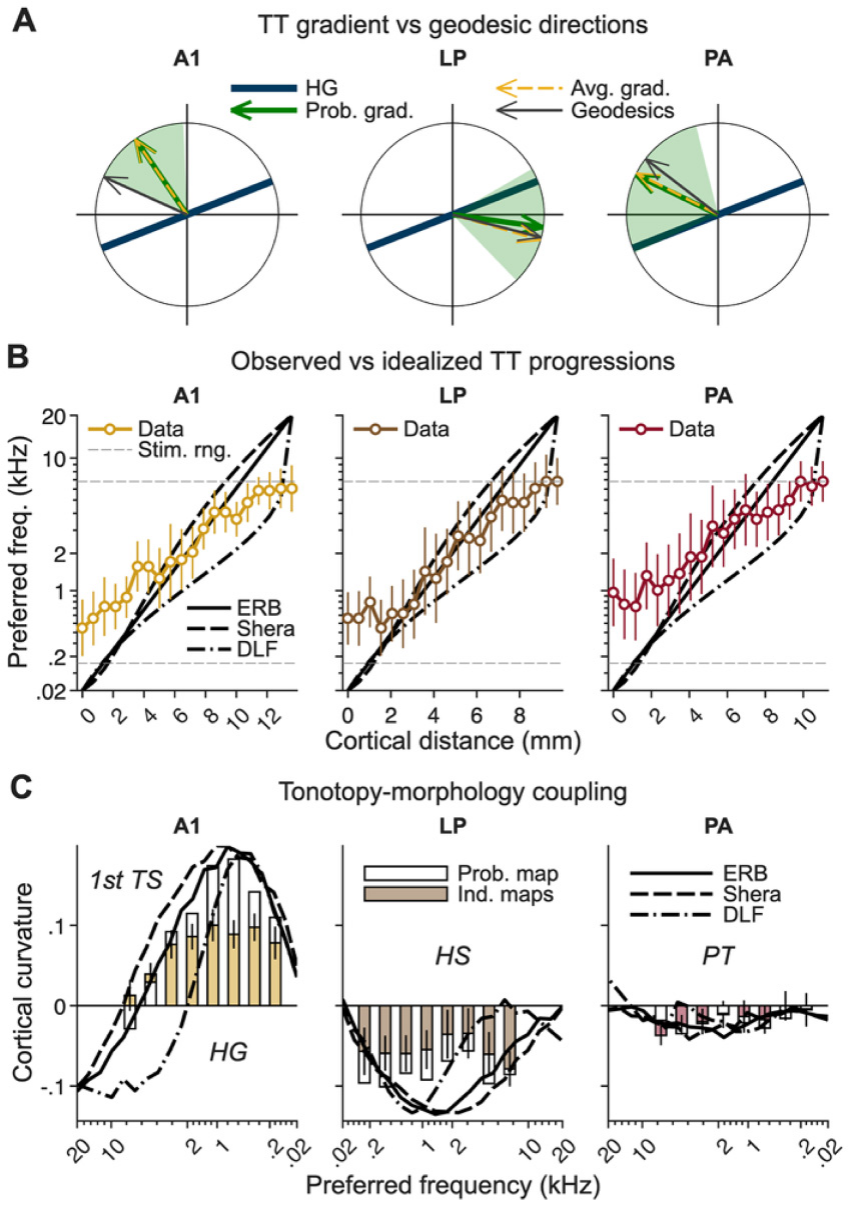
Comparison of fMRI tonotopy data and idealized models within A1, LP, and PA. (A) Area-average directions of geodesic paths (dark gray arrows) and tonotopic (TT) gradients. Green arrows: probabilistic TT gradients; dashed yellow arrows: average individual TT gradients; green shading: circular 95% CIs. The thick dark-blue line shows the HG long axis, estimated from cortical curvature gradients within A1. (B) Tonotopic progressions along geodesic paths; colored symbol/lines: average probabilistic preferred frequencies with bootstrap 95% CIs (error bars); bold black lines: idealized preferred-frequency progressions, both averaged within 20 equal-width bins. Abscissas show realistic cortical distances from the progressions’ low-frequency endpoints (measured on the 3D cortical surface). Gray dashed lines: fMRI stimulus range. (C) Vertex-wise cortical curvature versus preferred frequency for individual hemispheres (colored bars, with 95% CIs) and the Colin27 brain (white bars). Bold black lines: idealized tonotopy models (ERB, Shera, and DLF). 1st TS: first transverse sulcus; HG: Heschl's gyrus; HS: Heschl's sulcus; PT: planum temporale.

In areas LP and PA (Figure 7A, middle and right panels), the average geodesic orientation closely matched the mean of the individual area-average tonotopic gradient directions (z ≤ 1.190, *P* ≥ .468), which, in turn, agreed with the area-average gradient directions derived from the probabilistic tonotopic map. In contrast, in area A1, the average individual and probabilistic tonotopic gradients pointed significantly more medially than the geodesics (z = 4.437, *P* < .001; compare green/yellow and dark-gray arrows in Figure 7A, left panel). This difference could reflect a true discrepancy between geodesic and tonotopic gradient directions, but it might also reflect a systematic bias in the estimated tonotopic gradients within A1. The fMRI tonotopy data were acquired for frequencies ranging from 0.16 to 6.8 kHz (*Methods: MRI Data Acquisition and Analysis—Image Acquisition*), which is considerably narrower than the full audible range (20 Hz–20 kHz). Vertices with true preferred frequencies outside the stimulus range would therefore be incorrectly assigned to the stimulus range's nearest edge (0.16 or 6.8 kHz). As a result, tonotopic gradient strengths within the H_1_ and L_1_ high- and low-frequency regions would be close to zero, resulting in poorly defined gradient directions. Because of the wedge-like shape of the L_1_ region (Figure 6A and 6B), this may have caused the estimated gradients there to incorrectly point posteromedially along the HG long axis, whilst actual gradients likely point anteriorly toward H_1_. As a result, the area-average estimated gradient direction for A1 would be biased toward pointing too medially.

The probabilistic tonotopic mapping data (Figure 7B, colored symbols and lines) suggested that the shapes of idealized frequency progressions—here referred to as “frequency-position functions” in analogy to cochlear terminology (Greenwood, 1990)—should be similar across areas. As the cortical frequency-position function in humans remains unknown, a rational assumption could be that it resembles the cochlear function. Based on this assumption, equal cortical distances would represent equally resolvable frequency differences. Here, we tested two versions of this assumption: one based on estimates of equivalent rectangular cochlear filter bandwidth (ERB) from psychophysical notched-noise masking (the “ERB model”; Glasberg & Moore, 1990) and another based on estimates of cochlear frequency resolution from otoacoustic emission latencies (the “Shera model”; Shera et al., 2002; see *Methods: Forward Simulations—Construction of Idealized Tonotopic Maps*). Figure 7B shows that both models closely matched the observed frequency progressions (based on the probabilistic preferred frequencies) near the progression midpoints. Discrepancies near the progression edges are expected because the assumed tonotopic map limits (20 Hz and 20 kHz) differed from those of the stimulus-frequency range (0.16–6.8 kHz).

At the same time, both models consistently overestimated the progression slopes. To address this shortcoming, we additionally tested a model assuming that cortical tonotopic distances reflect, not frequency resolvability, which is determined in the cochlea (Evans et al., 1989; Evans et al., 1992; Sumner et al., 2018), but frequency discriminability, which is likely determined more centrally (Altes, 1989). Under this assumption, the slope of cortical frequency progressions is defined by the reciprocal of the frequency discrimination threshold, or frequency difference limen (DLF). DLFs are considerably smaller than frequency resolution thresholds at lower and middle frequencies (Micheyl et al., 2012). Consequently, the DLF-based frequency-position function initially rises more gradually than for the ERB- and Shera-models (compare black lines in Figure 7B), resulting in relatively expanded representations for lower and middle frequencies. Within the stimulus-frequency range, the DLF model closely matched the slopes of the observed (probabilistic) preferred-frequency progressions. At the same time, the model systematically underestimated the observed preferred-frequency values. These observed values, however, may have been biased upwards because lower-frequency stimuli were likely less salient than higher-frequency ones (see *Methods: MRI Data Acquisition and Analysis—Image Analysis)*. Supporting this interpretation, at the low-frequency endpoints of the progressions, average observed preferred frequencies did not reach the lower edge of the stimulus frequency range, whereas at the high-frequency endpoints, they consistently reached the upper edge (horizontal gray dashed lines in Figure 7B).

The Colin27 brain, used to construct the AER forward model (*Methods: Forward Simulations—EEG Forward Model*), features a single Heschl's gyrus in both hemispheres, whereas a proportion of the individual hemispheres in our fMRI tonotopy data set featured duplicated or partially duplicated gyri. Nevertheless, the relationship between vertex-wise probabilistic preferred frequencies and Colin27 cortical curvatures effectively captured the key characteristics of the corresponding relationships in individual hemispheres (based on individual preferred frequencies and cortical curvature values; compare white and colored bars in Figure 7C): in both cases, maximum positive curvature values—marking the HG crest within A1 in Colin27—were associated with preferred frequencies between 0.65 and 1.1 kHz; in LP, all preferred frequencies were consistently linked with negative curvature values, presumably marking Heschl's sulcus; and in PA, curvature values were consistently near zero, in accordance with a location on PT, which contains little or no gyrification. Among our idealized tonotopy models, the ERB model (solid black lines in Figure 7C) yielded the closest match to the observed frequency–curvature relationships, particularly in A1. In this model, the HG crest was occupied by frequencies around 0.77 kHz, with lower and higher frequencies represented on the gyrus's posterior and anterior banks, respectively. In the Shera model (dashed black lines), which expands the high-frequency representation relative to the ERB model, higher frequencies extended further toward the posterior bank of HG, placing the crest near frequencies around 1 kHz. In contrast, in the DLF model (dash-dotted black lines), which expands lower- and mid-frequency representations, lower frequencies were shifted toward the anterior bank of HG, so the gyrus crest was occupied by lower frequencies, around 0.56 kHz.

The pronounced differences in frequency-curvature relationships between A1, LP, and PA suggest that AER contributions from these areas should differ substantially in their amplitude–frequency dependence and general frequency-dependent properties. In the next section, we quantify these differences by combining the idealized tonotopy models with an EEG forward model to simulate frequency-specific AERs for each area.

#### Frequency Dependence of AER Contributions From Functional Auditory Areas

Using the idealized tonotopy models, we generated frequency-specific activation patterns for each functional area (A1, LP, and PA) at six octave-spaced stimulus frequencies spanning the range most represented in our meta-analysis (0.25–8 kHz). Each vertex was assigned an activation strength determined by the separation between its preferred frequency and the stimulus frequency (*Methods: Forward Simulations—Simulation of Neural Activation Patterns*). This step required assumptions about neural tuning widths, which cannot be directly measured in humans. Here, we estimated neural tuning widths from the widths of the area-average population receptive fields (pRFs) derived from our 7-T fMRI data (Figure 6C and 6E), and we also varied tuning width parametrically to systematically explore its effect on the simulated responses (discussed in the next section). The pRFs showed that frequency-specific fMRI activation patterns spanned a substantial proportion of the total tonotopic range: even in A1 and LP, pRF widths averaged 4.2 and 4.9 octaves on average (FWHM), corresponding to ∼42% and ∼49% of the total tonotopic range (20 Hz–20 kHz ≈ 9.97 octaves); in PA, average pRF width increased to 7.7 octaves, or ∼77% of the total tonotopic range. Part of these widths reflects the finite bandwidth (∼0.94 octaves) of the four-stimulus sets used in our fMRI analysis (see *Methods: MRI Data Acquisition and Analysis—Image Preprocessing and Analysis*), as well as the spatial spread of fMRI responses along tonotopic progressions. The latter is determined by the point spread function (PSF), which for GE-BOLD fMRI at 7 T ranges between 1 and 2 mm (Fracasso et al., 2021; Shmuel et al., 2007), and by the tonotopic gradient magnitude—the rate of frequency change along tonotopic progressions. Its effect on pRF width (as a fraction of the total tonotopic range) can be estimated by dividing the PSF (assumed as 1.5 mm) by the overall length of tonotopic progressions, that is, the area-average 3D geodesic path lengths, which measured 13.6 mm for A1, 9.75 mm for LP, and 11.0 mm for PA. After adjustment for both stimulus bandwidth and spatial spread, estimated neural tuning widths were reduced to 2.1, 2.4, and 5.4 octaves for A1, LP, and PA (corresponding to ∼21%, ∼24%, and ∼54% of the total tonotopic range; darker-shaded bars in Figure 6E, right panel).

**Figure 8.**
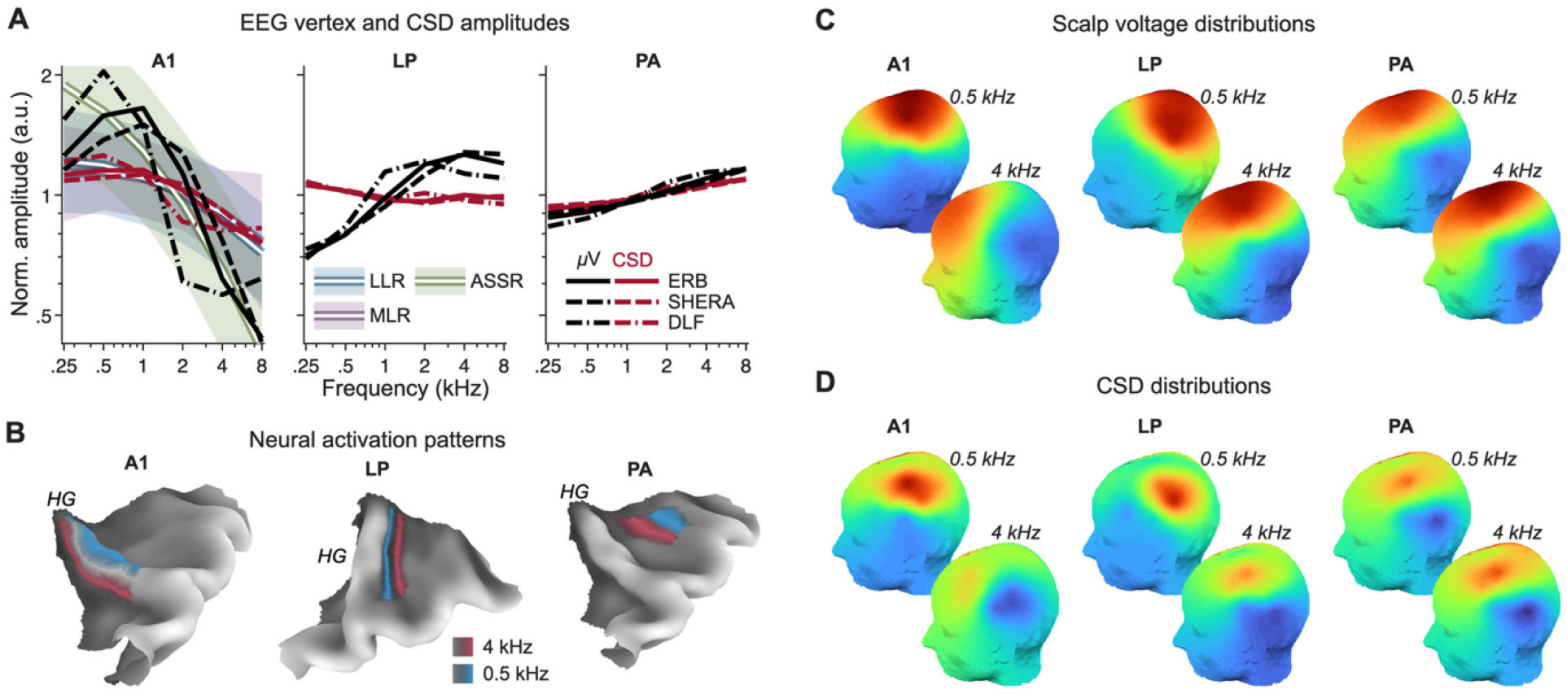
Simulated AER amplitudes and scalp topographies. (A) Simulated response amplitudes (bold black lines) from A1, LP, and PA, based on ERB, Shera, and DLF idealized tonotopy models (different line styles) and estimated neural tuning widths from Figure 6E. Colored lines and shading: observed amplitude–frequency relationships for the MLR, LLR, and 40-Hz ASSR, replotted from Figure 5. (B) Example simulated neural activation patterns for the 0.5- and 4-kHz stimulus frequencies and the ERB tonotopy model. (C) Scalp topographies for activation patterns in B. (D) CSDs of scalp topographies in C.

To simulate EEG response amplitudes, frequency-specific activation patterns (see Figure 8B for examples) were combined with the EEG forward model (head volume conductor and source model; Figure 2E) to generate scalp voltage distributions (Figure 8C), and, from these, response amplitudes (Figure 8A, bold black lines) were derived using the vertical montage (between vertex and mastoid electrodes), as employed by most EEG studies in our meta-analysis (*Results: Meta-Analysis*). The simulation results indicated that the shape of the amplitude–frequency dependence of auditory-evoked EEG responses should vary markedly with their cortical area of origin (Figure 8A). Across all three tonotopy models (ERB, Shera and DLF), A1 response amplitudes first increased slightly with frequency before dropping sharply toward higher frequencies (Figure 8A, left panel). The point of maximum response appeared to be determined by the frequency represented on the crest of HG, being lowest for the DLF model (0.5 kHz), intermediate for the ERB model (0.5–1 kHz) and highest for the Shera model (1 kHz). In contrast, LP and PA response amplitudes consistently increased with frequency, with PA showing a more uniform and slower rate of increase than LP. Comparison with the meta-analytic data from Figure 5 (replotted in Figure 8A) indicates that the rate of simulated amplitude decline for A1 closely matched that reported for the 40-Hz ASSR and was steeper than for either of the transient-evoked components (MLR and LLR). This suggests that the difference in the rate of decline of measured AER amplitudes between steady-state and transient-evoked responses may reflect a difference in these responses’ relative weight of A1 contributions.

**Figure 9.**
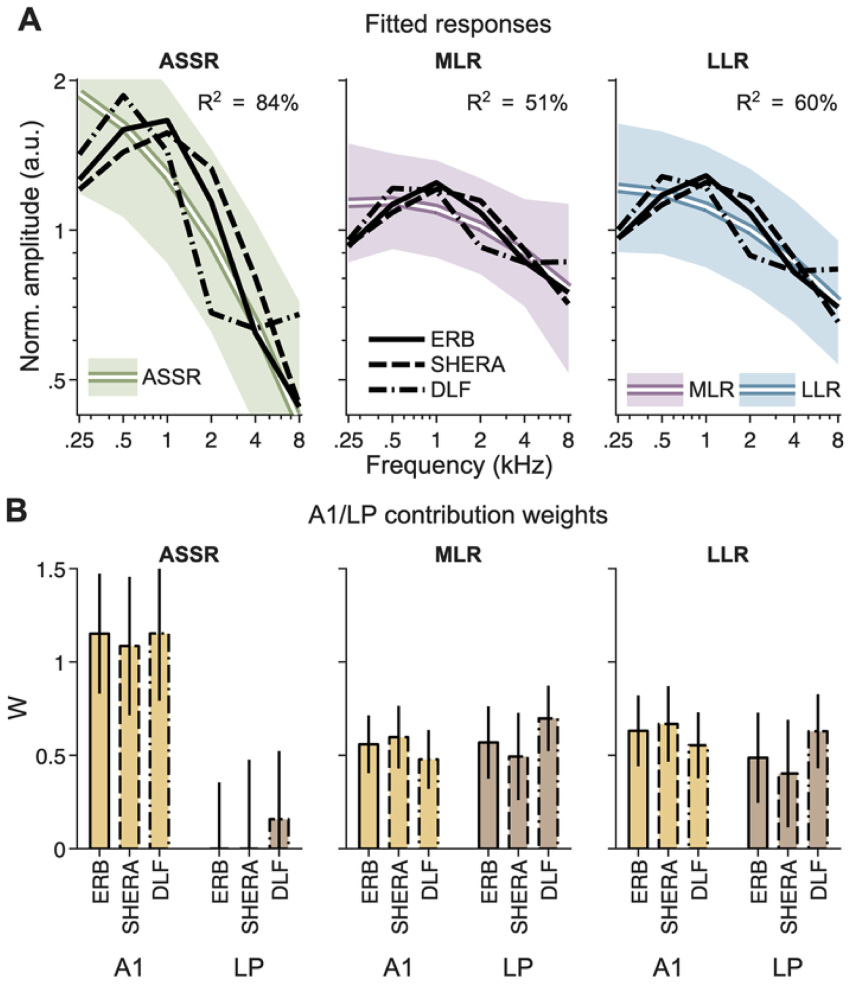
Estimation of areal contributions to transient-evoked and steady-state cortical AERs. (A) Observed AER amplitudes (colored lines/shading) with best-fitting simulated amplitudes (bold black lines) based on linear combinations of the simulated response contributions from A1 and LP (Figure 8A). Line styles: tonotopy models (ERB, Shera, and DLF). R^2^-values (explained variance in percent) are given for the ERB model, which was the best-fitting model across components. (B) Best-fitting contribution weights from A1 and LP; error bars: residual-bootstrap 95% CIs.

To test this assumption directly, we fitted the average reported amplitude–frequency relationship for each cortical AER component from our meta-analysis (LME fits shown in Figures 4 and 5) as a linear combination of the corresponding simulated relationships for areas A1 and LP (Figure 9A). Because the amplitude–frequency profiles of LP and PA are correlated (Figure 8A), fitting all three areas simultaneously (including PA) was not feasible with the available data, as the model would be under-constrained. Accordingly, the resulting fits should be interpreted as an illustrative demonstration rather than a quantitative estimate of true anatomical contributions. Similar results were obtained when using A1 and PA instead of A1 and LP in the fits (data not shown). Across all three tonotopy models (ERB, Shera and DLF), the best-fitting contribution weights were compatible with a strongly A1-dominated contribution to the 40-Hz ASSR, while the transient-evoked components (MLR and LLR) appeared to draw more comparable contributions from other areas (Figure 9B).

Panels C and D in Figure 8 indicate that frequency-related changes in simulated EEG response amplitudes are primarily driven by the orientation of the net neuroelectric current—often conceptualized as an equivalent current dipole—relative to the vertical axis along which amplitude is measured (vertical montage). As stimulus frequency increases, the net evoked current in A1 shifts from a slight posterior to a strong anterior tilt as neural activation moves from the top of the posterior bank of HG, across its crest, toward its anterior bank (see Figure 8B). In the scalp topographies (Figure 8C), this shift causes the central voltage maximum to move from slightly behind the vertex (Cz) channel at low frequencies to well in front of it at high frequencies, explaining why the simulated amplitudes first increase slightly and then decrease sharply with frequency. In contrast, the net current in LP shifts from a posterior tilt to a more vertical orientation as neural activation moves from the posterior bank of HG toward PT. This causes the central voltage maximum to shift toward the Cz channel from the rear, explaining why the simulated amplitudes from LP first increase with frequency and then reach a plateau. In contrast to A1 and LP, the increase in simulated amplitudes from PA was not accompanied by any notable change in scalp topography, suggesting that it reflects the area's tapered shape along the STP, which narrows toward its posterior end where lower frequencies are represented. Consequently, lower frequencies occupy less cortical surface area and thus elicit smaller net current moments than higher frequencies.

Thus, while simulated amplitude changes in A1 and LP were primarily driven by shifts in source orientation, amplitude changes in PA were mostly due to variations in source strength. The relative contributions of source orientation versus strength within each area can be illustrated by comparing the simulated EEG responses’ original amplitudes (μV across the vertical-montage) with the average (root-mean-square) amplitudes of their current source densities (CSDs, Figure 8D). CSDs reflect radial current flow occurring at the scalp surface and provide a direct, reference-free measure of the strengths of underlying neural currents (Kayser & Tenke, 2015). Figure 8A (bold red lines) shows that, in A1, changes in CSD amplitude follow the same trend as those of the original amplitudes (both decrease with frequency) but are much smaller in size. In LP, CSD amplitude changes are minimal and opposite to those in original amplitudes (CSD amplitudes decrease slightly, while original amplitudes increase). Finally, in PA, CSD and original amplitude changes not only follow the same direction but are also similar in magnitude. The CSD results suggest that the amplitude decreases observed in MEG studies are caused through related but partly distinct mechanisms compared to those in EEG studies. Specifically, if decrease in EEG amplitudes is caused by misalignment of the source orientation with the vertical recording montage, then the decrease in MEG amplitudes likely results from misalignment between the sensor array and the regions of strongest magnetic flux, driven by concomitant changes in source orientation and location. This implies that the rate of frequency-related amplitude decrease observed in MEG studies should depend on the size and configuration of the sensor array, with smaller arrays (linear or circular) showing a greater decrease than whole-head systems.

#### Effects of Tuning Width and Transversal Subdivision

Our initial simulations were based on neural tuning width estimates derived from fMRI population receptive-field widths, and on the assumption that each tonotopic gradient corresponds to a single functional area (the so-called “one map, one area” assumption). These assumptions, however, may be questionable. In particular, EEG and fMRI sources may differ in tuning properties: whilst fMRI reflects total synaptic contrast energy, EEG is sensitive to the spatial variance, synchrony, and polarity of synaptic currents—a difference that may profoundly impact these responses’ overall tuning selectivity (Hermes et al., 2019; Hermes & Siero, 2022). To assess the extent to which the initial simulations depended on these assumptions, we next examined how the simulated responses would change when tuning width is systematically varied—from extremely narrow, spanning only a small fraction (5%) of the tonotopic axis, to very broad, covering a large proportion (80%) of its overall range. In addition, we also relaxed the “one map, one area” assumption. Post-mortem cytoarchitectonic data (Morosan et al., 2001) suggest that the human primary auditory cortex (represented by the two most anterior of our three tonotopic gradients, A1 and LP) may be further subdivided transversally (parallel to the tonotopic axis) into multiple areas, or bands (TE1.0, TE1.1, TE1.2; Figure 3A), at least two of which (TE1.0 and TE1.1) exhibit primary-like features. This implies that a single tonotopic gradient may comprise distinct sub-regions with different neural properties. To specifically test the potential influence of such subdivisions, we implemented a simplified version of this organization by dividing each of our modeled tonotopic gradients (A1, LP, and PA) into medial and lateral halves (Figure 3B). These subdivisions were not intended as neurobiologically realistic parcellations—current anatomical evidence remains too limited to support specific transverse boundaries—but were instead meant to illustrate, in principle, how the presence of transverse subdivision would affect the simulation results.

**Figure 10.**
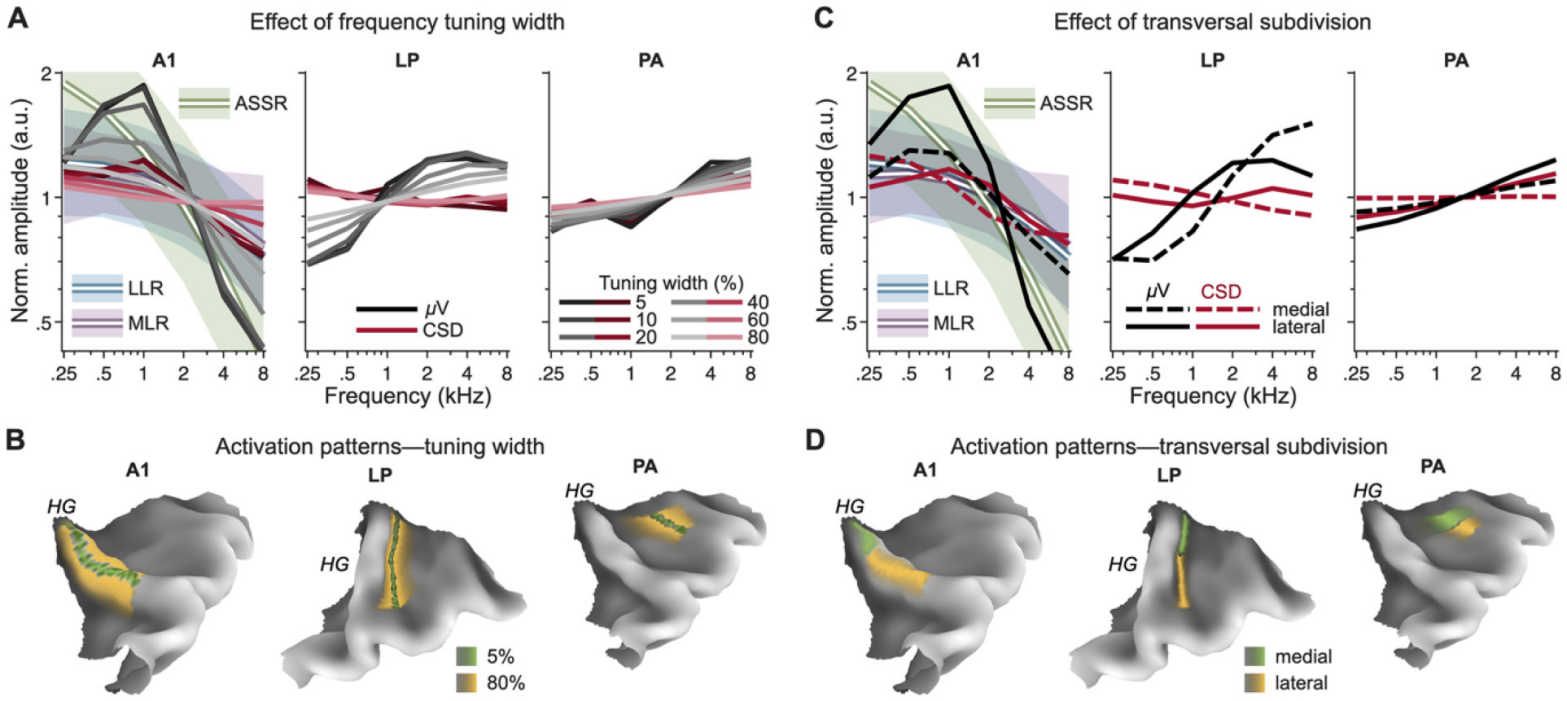
Effects of neural tuning width and transversal subdivision on simulated frequency-amplitude dependence of cortical AERs. (A) Simulated vertical-montage amplitudes (gray lines) and root-mean-square CSD amplitudes (red lines) for the ERB tonotopy model and neural tuning width varied parametrically from 5% to 80% of the total tonotopic range (20 Hz–20 kHz; lighter shades: greater tuning widths). Colored lines/shading: observed amplitude–frequency relationships for the MLR, LLR, and 40-Hz ASSR. (B) Example simulated activation patterns for the most extreme tuning widths of 5% (green) and 80% (yellow) at a 1-kHz stimulus frequency. (C) Effect of transversally subdividing each functional area (A1, LP, and PA) into medial (dashed lines) and lateral (solid lines) halves, using the ERB tonotopy model and pRF-based tuning width estimates. (D) Simulated medial (green) and lateral (yellow) activation patterns for a 1-kHz stimulus frequency.

The effect of variation in tuning width is illustrated in Figure 10A (different-shaded lines). The most pronounced effect is apparent in the simulated original (vertical montage) amplitudes (μV) of A1 and, to a lesser extent, LP, where broader tuning was associated with shallower frequency-related amplitude changes. CSD amplitudes were much less affected by tuning width variation than the original amplitudes. Overall, the qualitative differences between areas, and between original and CSD amplitudes, remained stable despite the large range of tuning widths tested.

The same was also true when activation patterns were restricted to either the medial or the lateral half of each functional area (compare dashed and solid lines in Figure 10C). The greatest effect of medial versus lateral source location was apparent in A1, where original amplitudes for medial sources (black dashed line) showed a considerably smaller frequency-related decline than for lateral sources (black solid line), whereas CSD amplitudes—conversely—showed a slightly greater decline (red dashed and solid lines). This reversal may be due to the smaller width of HG and, probably relatedly, the central low-frequency region, L_1_, at the gyrus's medial compared to lateral end (Figure 6A and 6B). As a result, frequency-specific activation patterns span a greater range of cortical curvatures, diluting the curvature effect on source orientation and thus vertical-montage amplitude, whilst, at the same time, increasing the effect on source strength (net current moment), and thus CSD amplitude, because sources spanning a greater range of curvatures (particularly within the first transverse sulcus) are less “open” due to mutual cancelation of currents. These results raise a possible alternative explanation of the observed difference in frequency-related amplitude decline between 40-Hz ASSRs and transient-evoked AERs (MLR/LLR): ASSRs may predominantly originate from area TE1.0, located centrally on HG (Figure 3A; light green highlight). In contrast, one or both of the transient responses (MLR and/or LLR) may arise from TE1.1, situated at the gyrus's medial end (Figure 3A; light purple highlight).

## Discussion

Little is currently known about how cortical morphology and functional organization influence cortical AERs. Here, we provide evidence that effects of morphology on obligatory (exogenous) AERs, originating in unimodal (supratemporal) auditory cortex (Godey et al., 2001; Liegeois-Chauvel et al., 1994; Näätänen & Picton, 1987), are strongly shaped by the underlying tonotopic layout. Consistent with previous fMRI studies (reviewed in Besle et al., 2019; Saenz & Langers, 2014), we show that, in humans, the predominant tonotopic axis runs approximately parallel to the main axis of morphological variation, that is, perpendicular to the long axis of HG. A realistic forward model of AERs revealed that tonotopy-related changes in source morphology may be a key driver of frequency-related variation in cortical AER characteristics, including the widely observed decline in AER amplitude at higher frequencies (Tlumak et al., 2016)—an effect clearly and consistently confirmed by our meta-analysis.

The simulation results suggest that the primary cause of frequency-related amplitude decline in EEG responses, measured with the vertical montage, is a systematic change in net source orientation driven by shifts in neural activation along tonotopic progressions within the highly myelinated primary area, A1. As stimulus frequency increases, activation within A1 moves anteriorly from the posterior bank and crest of HG to the gyrus's anterior bank, causing net source orientation to tilt forward—away from the vertical recording montage. The resulting predicted changes in response scalp topography (Figure 8) cannot be verified from our meta-analytic data, which lack scalp topography information. Previous source localization studies of AERs, however, offer tentative support: although source orientation was rarely assessed systematically, several reports indicate an anticlockwise sagittal (forward) tilt with increasing stimulus frequency (Pantev et al., 1996; Rosburg et al., 1998; Tiitinen et al., 1993). For the LLR (N1/N1m), frequency-related changes in source orientation were generally small and sometimes inconsistent, with some studies indicating a predominantly coronal rather than sagittal tilt (Bertrand et al., 1991; Pantev et al., 1995). Tiitinen et al. (1993) reported similar sagittal tilts for both the N1m and mismatch negativity (MMNm) of approximately 5° per octave, implying a potential 25° tilt across the five-octave range (250 Hz−8 kHz) used in the current simulations. Results by Pantev et al. (1996) suggest that sagittal tilt in the 40-Hz ASSR may be even larger—up to 10° per octave, or roughly 50° across our simulated range. A greater tilt in the ASSR than LLR aligns with our meta-analytic findings that the ASSR exhibits a steeper frequency-related amplitude decline, and with the forward simulations suggesting that the ASSR receives predominant—perhaps exclusive—contributions from the lateral portion of A1, located on the central part of HG.

In contrast, the shallower amplitude–frequency relationships of the LLR and—perhaps more unexpectedly—the MLR (Pa/Pam) suggest that these components may receive additional contributions from at least one other area, likely LP, which both our and previous fMRI data (Besle et al., 2019) indicate is part of the auditory primary region. Given that A1 and LP exhibit mirror-symmetric tonotopic maps, this may explain why EEG and MEG studies of cortical tonotopy—most of which have focussed on the N1/N1m—have consistently failed to identify a coherent tonotopic axis (reviewed in Lütkenhöner et al., 2003b). The idea that the Pa/Pam—the first cortical AER deflection—receives contributions from areas other than A1 may seem surprising, but is consistent with findings by (Lütkenhöner et al., 2003a) who employed high-precision, single-subject MEG source mapping to show that the source topography of the MLR changes rapidly after the Pa/Pam reaches its peak. To localize A1, they analyzed the rising flank of the Pam, referred to as dP20m, and found that the dP20m and Pam peaks do not share the same cortical origin. Specifically, the Pam appeared to receive major contributions from at least two distinct cortical sources, only one of which was A1.

In contrast, the idea that ASSRs may be restricted to A1 is supported by fMRI evidence indicating that primary auditory areas—similar to subcortical structures—show sustained (tonic) responses to prolonged, rapidly amplitude-modulated sounds such as those used to elicit 40-Hz ASSRs, whereas non-primary areas show phasic responses, primarily limited to the sound onset and offset (Harms et al., 2005; Harms & Melcher, 2002, 2003; Seifritz et al., 2002). These differences imply progressively longer adaptation recovery times along the auditory hierarchy, which may underlie the more complex adaptation phenomena observed at higher processing levels (Perez-Gonzalez & Malmierca, 2014) and/or support adaptation-based neural coding efficiency (Mlynarski & Hermundstad, 2021).

The current functional parcellation of the supratemporal auditory region into A1, LP, and PA follows the “one map, one area” assumption, widely applied in the visual domain, where retinotopic maps are presumed to define distinct visual areas (Sereno et al., 1995; Wandell et al., 2007). Consistent with our previous findings (Besle et al., 2019), obtained using different subjects and tonotopy stimuli, we observed both A1 and LP to exhibit primary-like qualities, with A1 showing elevated intra-cortical myelin content, and both areas showing increased frequency selectivity, reflected in narrower pRF tuning widths. These features suggest that A1 and LP correspond to the histochemically defined areas described by Wallace et al. (2002). Similar to those histochemical areas, the functional areas extended across approximately two-thirds of the posteromedial length of HG.

Cytoarchitectonic findings (Morosan et al., 2001) have indicated that A1 and potentially also LP may be subdivided transversally, that is, perpendicular to its long axis. Such subdivisions would run parallel to the predominant tonotopic gradients and would therefore not be marked by gradient reversals. The cytoarchitectonic data identified three subdivisions, TE1.0, TE1.1, and TE1.2. Of these, TE1.0, located in the central portion of HG, is considered the most primary area for showing the thickest granular layer IV. TE1.1, positioned posteromedially to TE1.0, exhibits less distinct laminar differentiation, while TE1.2 shows features typical of non-primary auditory cortex. Because TE1.2 occupies the anterolateral part of HG, it is unlikely to have been substantially included in our functionally defined areas. Accordingly, TE1.0 and TE1.1 may approximately correspond to the lateral and medial portions of areas A1 and LP (Figure 3). Forward simulations indicated that responses arising from the medial part of A1 should exhibit a shallower amplitude–frequency slope than responses arising from the lateral part, offering a potential alternative explanation of the observed slope difference between transient-evoked AERs (MLR/LLR) and the 40-Hz ASSR. However, we emphasize that, while there is strong evidence, across sensory modalities, that functionally defined areal borders (i.e., functional gradient reversals) coincide with structural boundaries (Besle et al., 2019; Hinds et al., 2009; Sanchez-Panchuelo et al., 2012), there is currently no in-vivo evidence supporting a transverse subdivision of A1 and/or LP into more primary lateral and less primary medial parts, and such a subdivision is also not supported by the majority of existing post-mortem evidence (reviewed in Baumann et al., 2013). In fact, both our (Figure 6C) and previous in-vivo MRI data on intra-cortical myelin content (Besle et al., 2019; Dick et al., 2012; Sigalovsky et al., 2006) suggest higher myelination in the posteromedial than central portion of HG (note, however, that this may, in part, reflect insufficiently corrected artefactual effects of cortical curvature and thickness; Besle et al., 2019; Sereno et al., 2013; Tzourio-Mazoyer et al., 2019).

To test how strongly the predicted frequency-related changes in simulated AER amplitudes depend on the modeling assumptions, we used three alternative cortical magnification schemes—two based on cochlear frequency–position functions (the ERB and Shera models), and one assuming an expanded low-frequency representation relevant for speech and music (the DLF model; Altes, 1989). Despite substantial differences in the implied relationship between local preferred frequency and local cortical curvature (Figure 7C), the three schemes yielded qualitatively and even quantitatively similar predictions of scalp-level amplitude–frequency dependence for all three cortical areas tested (A1, LP, and PA). Likewise, varying neural tuning width—and thus the spatial extent of simulated activation patterns—across an extensive range (from 5% to 80% of the total tonotopic span) produced qualitatively similar predictions of amplitude–frequency dependence. At the broadest tuning widths, however, the simulations failed to reproduce the steeper frequency-related amplitude decline of the 40-Hz ASSR, as the predicted relationships became uniformly shallow, even for A1 (Figure 10A). This behavior suggests another possible explanation for why transient-evoked AER components (MLR/LLR) show a shallower amplitude–frequency dependence than steady-state responses (40-Hz ASSR): if one or both transient components reflect feedback-dominated rather than feedforward drive, and therefore exhibit broader underlying tuning, the resulting scalp responses would be expected to vary less steeply with frequency, even if all responses arise from the same general cortical region.

Another key assumption of our simulations was that all stimulation frequencies elicited the same density of neuro-electric currents, and thus the same net current per unit cortical area (*Methods: Forward Simulations—Simulation of Neural Activation Patterns*). This assumption was necessary to isolate the effect of the geometrical configuration of cortical source distributions. In reality, however, cortical current density may vary systematically with stimulation frequency. If such frequency-dependent differences in physiological activation strength exist, they would introduce an additional source of amplitude variation across frequencies that is not captured by the present model.

The current study has many limitations and can only represent a first step toward understanding how tonotopic organization shapes the source morphology of AERs and how, in turn, this influences their observed scalp characteristics. Firstly, the meta-analytic data lacks information on scalp distributions of responses. Most studies reporting frequency-dependent AER amplitudes date from an era when EEG recordings used few electrodes and data sharing was uncommon. Consequently, we cannot test a key prediction of our forward model: that increasing stimulus frequency tilts the net source orientation forward and that this at least partially explains the frequency dependence of AER amplitudes. The lack of scalp topography data also limits predictions about relationships between EEG and MEG. Our forward simulations suggest that morphology-related reductions in AER amplitude arise from two factors. The first is misalignment between response scalp topography and sensor layout. For EEG, this means mismatch between source orientation and the vertical montage. For MEG, by contrast, it means misalignment between regions of peak magnetic flux and the sensor array—an effect that should vary with sensor layout (e.g., linear or circular vs whole-head systems). The second factor, estimated by our CSD analysis, is that the net current moment of underlying sources is smaller for responses originating from regions with high cortical curvature (particularly sulci) than from flatter regions. This factor should similarly affect both EEG and MEG responses and be independent of the sensor layout. Finally, our functional parcellation may not capture the entirety of the tonotopically organized supratemporal region contributing to relevant obligatory AERs and—more importantly—our forward simulations do not account for individual variation in auditory cortical morphology. Our simulations used a realistic brain template (Colin27; Aubert-Broche et al., 2006) with a single Heschl's gyrus in both hemispheres. Although this represents the most common gyrification pattern (Marie et al., 2015), variants involving HG duplication—particularly on the right—are also frequent. Predicting AERs for such patterns will require a better understanding of how gyrification affects tonotopic map layout (Besle et al., 2019; Da Costa et al., 2011). Alternatively, individual fMRI tonotopy data could be combined with corresponding individual AER recordings. If fMRI and AER data were acquired using the same stimuli, this would also eliminate the need for idealized tonotopy models and associated assumptions.

## Conclusions

Our results confirm that human tonotopic gradients align with the main axis of morphological variation in supratemporal auditory cortex, indicating that tonotopy should play a central role in shaping AER source morphology and, consequently, influencing response characteristics. Using tonotopic gradient reversals to identify areal borders, we parcellate the region into three areas—A1, LP, and PA—and show that A1 and LP are likely primary, whereas PA is non-primary. Our meta-analysis revealed significant differences in the slope of the amplitude–frequency relationship between steady-state (40-Hz ASSR) and transient-evoked responses (MLR, LLR). A realistic forward model of AERs suggested that these differences may relate to variation in the strength of the responses’ relative contribution from A1 compared to the other areas, or to differences in the relative contributions of medial versus lateral portions of A1, which may exhibit partially distinct response profiles.

## Supplemental Material

sj-pdf-1-tia-10.1177_23312165261454531 - Supplemental material for Reinterpreting the Frequency Dependence of Cortical Auditory-Evoked Response Amplitudes in Light of Current Understanding of Cortical Tonotopic Organization Supplemental material, sj-pdf-1-tia-10.1177_23312165261454531 for Reinterpreting the Frequency Dependence of Cortical Auditory-Evoked Response Amplitudes in Light of Current Understanding of Cortical Tonotopic Organization by Carl Rushworth, Alexander J. Hardy, Magdalena Sereda, Ben J. Gurer, Julien Besle, Susan T. Francis, Rebecca S. Dewey, Denis Schluppeck, Justin M. Ales, Vassilis Pelekanos, Michael A. Akeroyd and Katrin Krumbholz in Trends in Hearing

## References

[bibr1-23312165261454531] Abdul-Kareem I. A. Sluming V. (2008). Heschl gyrus and its included primary auditory cortex: Structural MRI studies in healthy and diseased subjects. Journal of Magnetic Resonance Imaging, 28(2), 287–299. 10.1002/jmri.21445 18666141

[bibr2-23312165261454531] Ales J. M. Carney T. Klein S. A. (2010a). The folding fingerprint of visual cortex reveals the timing of human V1 and V2. Neuroimage, 49(3), 2494–2502. 10.1016/j.neuroimage.2009.09.022 19778621 PMC2818454

[bibr3-23312165261454531] Ales J. M. Yates J. L. Norcia A. M. (2010b). V1 is not uniquely identified by polarity reversals of responses to upper and lower visual field stimuli. Neuroimage, 52(4), 1401–1409. 10.1016/j.neuroimage.2010.05.016 20488247 PMC2922686

[bibr4-23312165261454531] Altes R. A. (1989). Ubiquity of hyperacuity. The Journal of the Acoustical Society of America, 85(2), 943–952. 10.1121/1.397566 2926010

[bibr5-23312165261454531] Antinoro F. Skinner P. H. Jones J. J. (1969). Relation between sound intensity and amplitude of the AER at different stimulus frequencies. Journal of the Acoustical Society of America, 46(6), 1433–1436. 10.1121/1.1911881 5361509

[bibr6-23312165261454531] Arlinger S. Elberling C. Bak C. Kofoed B. Lebech J. Saermark K. (1982). Cortical magnetic fields evoked by frequency glides of a continuous tone. Electroencephalography and Clinical Neurophysiology, 54(6), 642–653. 10.1016/0013-4694(82)90118-3 6183097

[bibr7-23312165261454531] Aubert-Broche B. Evans A. C. Collins L. (2006). A new improved version of the realistic digital brain phantom. Neuroimage, 32(1), 138–145. 10.1016/j.neuroimage.2006.03.052 16750398

[bibr8-23312165261454531] Auerbach B. D. Gritton H. J. (2022). Hearing in complex environments: Auditory gain control, attention, and hearing loss. Frontiers in Neuroscience, 16, 799787. 10.3389/fnins.2022.799787 35221899 PMC8866963

[bibr9-23312165261454531] Baillet S. (2022). Forward and Inverse Problems of MEG/EEG. In Jaeger D. Jung R. (Eds.), Encyclopedia of computational neuroscience (pp. 1464–1471). Springer. 10.1007/978-1-0716-1006-0_529

[bibr10-23312165261454531] Bates D. Mächler M. Bolker B. Walker S. (2015). Fitting linear mixed-effects models using lme4. Journal of Statistical Software, 67(1), 1–48. 10.18637/jss.v067.i01

[bibr11-23312165261454531] Baumann S. Petkov C. I. Griffiths T. D. (2013). A unified framework for the organization of the primate auditory cortex. Frontiers in Systems Neuroscience, 7, 11. 10.3389/fnsys.2013.00011 23641203 PMC3639404

[bibr12-23312165261454531] Berens P. (2009). Circstat: A MATLAB toolbox for circular statistics. Journal of Statistical Software, 31(10), 1–21. 10.18637/jss.v031.i10

[bibr13-23312165261454531] Bertrand O. Perrin F. Pernier J. (1991). Evidence for a tonotopic organization of the auditory cortex observed with auditory evoked potentials. Acta Oto-Laryngologica, 111(sup491), 116–123. 10.3109/00016489109136788 1814142

[bibr14-23312165261454531] Besle J. Mougin O. Sanchez-Panchuelo R. M. Lanting C. Gowland P. Bowtell R. , …, & Krumbholz K. (2019). Is human auditory cortex organization compatible with the monkey model? Contrary evidence from ultra-high-field functional and structural MRI. Cerebral Cortex, 29(1), 410–428. 10.1093/cercor/bhy267 30357410 PMC6294415

[bibr15-23312165261454531] Cottereau B. R. Ales J. M. Norcia A. M. (2015). How to use fMRI functional localizers to improve EEG/MEG source estimation. Journal of Neuroscience Methods, 250, 64–73. 10.1016/j.jneumeth.2014.07.015 25088693 PMC4383720

[bibr16-23312165261454531] Da Costa S. van der Zwaag W. Marques J. P. Frackowiak R. S. Clarke S. Saenz M. (2011). Human primary auditory cortex follows the shape of Heschl's gyrus. Journal of Neuroscience, 31(40), 14067–14075. 10.1523/JNEUROSCI.2000-11.2011 21976491 PMC6623669

[bibr17-23312165261454531] Daniel P. M. Whitteridge D. (1961). The representation of the visual field on the cerebral cortex in monkeys. The Journal of Physiology, 159(2), 203–221. 10.1113/jphysiol.1961.sp006803 13883391 PMC1359500

[bibr18-23312165261454531] Davis H. Bowers C. Hirsh S. K. (1968). Relations of the human vertex potential to acoustic input: Loudness and masking. Journal of the Acoustical Society of America, 43(3), 431–438. 10.1121/1.1910849 5640948

[bibr19-23312165261454531] Delorme A. Makeig S. (2004). EEGLAB: An open source toolbox for analysis of single-trial EEG dynamics including independent component analysis. Journal of Neuroscience Methods, 134(1), 9–21. 10.1016/j.jneumeth.2003.10.009 15102499

[bibr20-23312165261454531] Dick F. Tierney A. T. Lutti A. Josephs O. Sereno M. I. Weiskopf N. (2012). In vivo functional and myeloarchitectonic mapping of human primary auditory areas. Journal of Neuroscience, 32(46), 16095–16105. 10.1523/JNEUROSCI.1712-12.2012 23152594 PMC3531973

[bibr21-23312165261454531] Dimitrijevic A. John M. S. Van Roon P. Purcell D. W. Adamonis J. Ostroff J. , …, & Picton T. W. (2002). Estimating the audiogram using multiple auditory steady-state responses. Journal of the American Academy of Audiology, 13(4), 205–224. https://www.ncbi.nlm.nih.gov/pubmed/12025896. https://doi.org/10.1055/s-0040-1715964 12025896

[bibr22-23312165261454531] Dumoulin S. O. Wandell B. A. (2008). Population receptive field estimates in human visual cortex. Neuroimage, 39(2), 647–660. 10.1016/j.neuroimage.2007.09.034 17977024 PMC3073038

[bibr23-23312165261454531] Eggermont J. J. Don M. (1980). Analysis of the click-evoked brainstem potentials in humans using high-pass noise masking. II. Effect of click intensity. Journal of the Acoustical Society of America, 68(6), 1671–1675. 10.1121/1.385199 7462466

[bibr24-23312165261454531] Elberling C. Bak C. Kofoed B. Lebech J. Saermark K. (1982). Auditory magnetic fields: Source location and “tonotopical organization” in the right hemisphere of the human brain. Scandinavian Audiology, 11(1), 61–65. 10.3109/01050398209076201 7178805

[bibr25-23312165261454531] Evans E. Pratt S. Cooper N. (1989). Correspondence between behavioural and physiological frequency selectivity in the Guinea pig. Br. J. Audiol, 23(2), 151. 10.1016/B978-0-08-041847-6.50024-1

[bibr26-23312165261454531] Evans E. Pratt S. Spenner H. Cooper N. (1992). Comparisons of physiological and behavioural properties: Auditory frequency selectivity. In Auditory physiology and perception (pp. 159–169). Elsevier. 10.1016/C2013-0-03863-7

[bibr27-23312165261454531] Evans T. R. Deatherage B. H. (1969). The effect of frequency on the auditory evoked response. Psychonomic Science, 15(2), 95–96. https://link.springer.com/article/10.3758/BF03336219. https://doi.org/10.3758/BF03336219

[bibr28-23312165261454531] Fischl B. (2012). Freesurfer. Neuroimage, 62(2), 774–781. 10.1016/j.neuroimage.2012.01.021 22248573 PMC3685476

[bibr29-23312165261454531] Fracasso A. Dumoulin S. O. Petridou N. (2021). Point-spread function of the BOLD response across columns and cortical depth in human extra-striate cortex. Progress in Neurobiology, 202, 102034. 10.1016/j.pneurobio.2021.102034 33741401 PMC8958221

[bibr30-23312165261454531] Fujioka T. Kakigi R. Gunji A. Takeshima Y. (2002). The auditory evoked magnetic fields to very high frequency tones. Neuroscience, 112(2), 367–381. 10.1016/s0306-4522(02)00086-6 12044454

[bibr31-23312165261454531] Gabriel D. Veuillet E. Ragot R. Schwartz D. Ducorps A. Norena A. , …, & Collet L. (2004). Effect of stimulus frequency and stimulation site on the N1m response of the human auditory cortex. Hearing Research, 197(1-2), 55–64. 10.1016/j.heares.2004.07.015 15504604

[bibr32-23312165261454531] Galambos R. Makeig S. Talmachoff P. J. (1981). A 40-hz auditory potential recorded from the human scalp. Proceedings of the National Academy of Sciences of the United States of America, 78(4), 2643–2647. 10.1073/pnas.78.4.2643 6941317 PMC319406

[bibr33-23312165261454531] Glasberg B. R. Moore B. C. (1990). Derivation of auditory filter shapes from notched-noise data. Hearing Research, 47(1-2), 103–138. 10.1016/0378-5955(90)90170-t 2228789

[bibr34-23312165261454531] Godey B. Schwartz D. de Graaf J. B. Chauvel P. Liegeois-Chauvel C. (2001). Neuromagnetic source localization of auditory evoked fields and intracerebral evoked potentials: A comparison of data in the same patients. Clinical Neurophysiology, 112(10), 1850–1859. 10.1016/s1388-2457(01)00636-8 11595143

[bibr35-23312165261454531] Gold J. R. Bajo V. M. (2014). Insult-induced adaptive plasticity of the auditory system. Frontiers in Neuroscience, 8, 110. 10.3389/fnins.2014.00110 24904256 PMC4033160

[bibr36-23312165261454531] Gramfort A. Luessi M. Larson E. Engemann D. A. Strohmeier D. Brodbeck C. , …, & Hamalainen M. (2013). MEG and EEG data analysis with MNE-python. Frontiers in Neuroscience, 7, 267. 10.3389/fnins.2013.00267 24431986 PMC3872725

[bibr37-23312165261454531] Greenwood D. D. (1990). A cochlear frequency-position function for several species – 29 years later. Journal of the Acoustical Society of America, 87(6), 2592–2605. 10.1121/1.399052 2373794

[bibr38-23312165261454531] Guiraud J. Besle J. Arnold L. Boyle P. Giard M. H. Bertrand O. , …, & Collet L. (2007). Evidence of a tonotopic organization of the auditory cortex in cochlear implant users. Journal of Neuroscience, 27(29), 7838–7846. 10.1523/JNEUROSCI.0154-07.2007 17634377 PMC6672887

[bibr39-23312165261454531] Hackett T. A. Preuss T. M. Kaas J. H. (2001). Architectonic identification of the core region in auditory cortex of macaques, chimpanzees, and humans. Journal of Comparative Neurology, 441(3), 197–222. 10.1002/cne.1407 11745645

[bibr40-23312165261454531] Hagler D. J. Jr. Halgren E. Martinez A. Huang M. Hillyard S. A. Dale A. M. (2009). Source estimates for MEG/EEG visual evoked responses constrained by multiple, retinotopically-mapped stimulus locations. Human Brain Mapping, 30(4), 1290–1309. 10.1002/hbm.20597 18570197 PMC2754810

[bibr41-23312165261454531] Hahn A. D. Nencka A. S. Rowe D. B. (2009). Improving robustness and reliability of phase-sensitive fMRI analysis using temporal off-resonance alignment of single-echo timeseries (TOAST). Neuroimage, 44(3), 742–752. 10.1016/j.neuroimage.2008.10.001 18992826 PMC2884970

[bibr42-23312165261454531] Harms M. P. Guinan J. J. Jr. Sigalovsky I. S. Melcher J. R. (2005). Short-term sound temporal envelope characteristics determine multisecond time patterns of activity in human auditory cortex as shown by fMRI. Journal of Neurophysiology, 93(1), 210–222. 10.1152/jn.00712.2004 15306629

[bibr43-23312165261454531] Harms M. P. Melcher J. R. (2002). Sound repetition rate in the human auditory pathway: Representations in the waveshape and amplitude of fMRI activation. Journal of Neurophysiology, 88(3), 1433–1450. 10.1152/jn.2002.88.3.1433 12205164

[bibr44-23312165261454531] Harms M. P. Melcher J. R. (2003). Detection and quantification of a wide range of fMRI temporal responses using a physiologically-motivated basis set. Human Brain Mapping, 20(3), 168–183. 10.1002/hbm.10136 14601143 PMC1866291

[bibr45-23312165261454531] Hermes D. Petridou N. Kay K. N. Winawer J. (2019). An image-computable model for the stimulus selectivity of gamma oscillations. Elife, 8, e47035. 10.7554/eLife.47035 PMC6839904 31702552

[bibr46-23312165261454531] Hermes D. Siero J. C. W. (2022). Neuronal models for EEG–fMRI integration. In Mulert C. Lemieux L. (Eds.), EEG – fMRI: Physiological basis, technique, and applications (pp. 625–638). Springer International Publishing. 10.1007/978-3-031-07121-8_25

[bibr47-23312165261454531] Hinds O. Polimeni J. R. Rajendran N. Balasubramanian M. Amunts K. Zilles K. , …, & Triantafyllou C. (2009). Locating the functional and anatomical boundaries of human primary visual cortex. Neuroimage, 46(4), 915–922. 10.1016/j.neuroimage.2009.03.036 19328238 PMC2712139

[bibr48-23312165261454531] Holmes C. J. Hoge R. Collins L. Woods R. Toga A. W. Evans A. C. (1998). Enhancement of MR images using registration for signal averaging. Journal of Computer Assisted Tomography, 22(2), 324–333. 10.1097/00004728-199803000-00032 9530404

[bibr49-23312165261454531] Holt F. Ozdamar O. (2016). Effects of rate (0.3-40/s) on simultaneously recorded auditory brainstem, middle and late responses using deconvolution. Clinical Neurophysiology, 127(2), 1589–1602. 10.1016/j.clinph.2015.10.046 26639172

[bibr50-23312165261454531] Inverso S. A. Goh X. L. Henriksson L. Vanni S. James A. C. (2016). From evoked potentials to cortical currents: Resolving V1 and V2 components using retinotopy constrained source estimation without fMRI. Human Brain Mapping, 37(5), 1696–1709. 10.1002/hbm.23128 26870938 PMC6867263

[bibr51-23312165261454531] Jacobson G. P. Lombardi D. M. Gibbens N. D. Ahmad B. K. Newman C. W. (1992). The effects of stimulus frequency and recording site on the amplitude and latency of multichannel cortical auditory evoked potential (CAEP) component N1. Ear and Hearing, 13(5), 300–306. 10.1097/00003446-199210000-00007 1487089

[bibr52-23312165261454531] Jenkinson M. Beckmann C. F. Behrens T. E. J. Woolrich M. W. Smith S. M. (2012). FSL. Neuroimage, 62(2), 782–790. 10.1016/j.neuroimage.2011.09.015 21979382

[bibr53-23312165261454531] Jewett D. L. Williston J. S. (1971). Auditory-evoked far fields averaged from the scalp of humans. Brain, 94(4), 681–696. 10.1093/brain/94.4.681 5132966

[bibr54-23312165261454531] John M. S. Dimitrijevic A. van Roon P. Picton T. W. (2001). Multiple auditory steady-state responses to AM and FM stimuli. Audiology & Neuro-Otology, 6(1), 12–27. 10.1159/000046805 11173772

[bibr55-23312165261454531] Johnsrude I. S. Giraud A. L. Frackowiak R. S. (2002). Functional imaging of the auditory system: The use of positron emission tomography. Audiology & Neuro-Otology, 7(5), 251–276. 10.1159/000064446 12232496

[bibr56-23312165261454531] Kadah Y. M. Hu X. (1997). Simulated phase evolution rewinding (SPHERE): A technique for reducing B0 inhomogeneity effects in MR images. Magnetic Resonance in Medicine, 38(4), 615–627. 10.1002/mrm.1910380416 9324329

[bibr57-23312165261454531] Kayser J. (2009). Current source density (CSD) interpolation using spherical splines – CSD Toolbox (Version 1.1). New York State Psychiatric Institute: Division of Cognitive Neuroscience. http://psychophysiology.cpmc.columbia.edu/Software/CSDtoolbox.

[bibr58-23312165261454531] Kayser J. Tenke C. E. (2006). Principal components analysis of Laplacian waveforms as a generic method for identifying ERP generator patterns: I. Evaluation with auditory oddball tasks. Clinical Neurophysiology, 117(2), 348–368. 10.1016/j.clinph.2005.08.034 16356767

[bibr59-23312165261454531] Kayser J. Tenke C. E. (2015). Issues and considerations for using the scalp surface Laplacian in EEG/ERP research: A tutorial review. International Journal of Psychophysiology, 97(3), 189–209. 10.1016/j.ijpsycho.2015.04.012 25920962 PMC4537804

[bibr60-23312165261454531] Kuwada S. Batra R. Maher V. L. (1986). Scalp potentials of normal and hearing-impaired subjects in response to sinusoidally amplitude-modulated tones. Hearing Research, 21(2), 179–192. 10.1016/0378-5955(86)90038-9 3700256

[bibr61-23312165261454531] Kuznetsova A. Brockhoff P. B. Christensen R. H. B. (2017). Lmertest package: Tests in linear mixed effects models. Journal of Statistical Software, 82(13), 1–26. https://doi.org/10.18637/jss.v082.i13. Retrieved from: https://cran.r-project.org/web/packages/lmerTest/index.html

[bibr62-23312165261454531] Lamberton F. Delcroix N. Grenier D. Mazoyer B. Joliot M. (2007). A new EPI-based dynamic field mapping method: Application to retrospective geometrical distortion corrections. Journal of Magnetic Resonance Imaging, 26(3), 747–755. 10.1002/jmri.21039 17729370

[bibr63-23312165261454531] Langers D. R. van Dijk P. (2012). Mapping the tonotopic organization in human auditory cortex with minimally salient acoustic stimulation. Cerebral Cortex, 22(9), 2024–2038. 10.1093/cercor/bhr282 21980020 PMC3412441

[bibr64-23312165261454531] Lenhardt M. L. (1971). Effects of the frequency of amplitude modulated signals on the auditory evoked response. Psychonomic Science, 24(1), 30–30. 10.3758/BF03331762

[bibr65-23312165261454531] Liegeois-Chauvel C. Musolino A. Badier J. M. Marquis P. Chauvel P. (1994). Evoked potentials recorded from the auditory cortex in man: Evaluation and topography of the middle latency components. Electroencephalography and Clinical Neurophysiology, 92(3), 204–214. 10.1016/0168-5597(94)90064-7 7514990

[bibr66-23312165261454531] Lins O. G. Picton T. W. Boucher B. L. Durieux-Smith A. Champagne S. C. Moran L. M. , …, & Savio G. (1996). Frequency-specific audiometry using steady-state responses. Ear and Hearing, 17(2), 81–96. 10.1097/00003446-199604000-00001 8698162

[bibr67-23312165261454531] Loy A. Steele S. Korobova J. (2023). lmeresampler: Bootstrap methods for nested linear mixed-effects models. https://CRAN.R-project.org/package=lmeresampler.

[bibr68-23312165261454531] Luck S. J. (2014). An Introduction to the Event-Related Potential Technique. MIT Press.

[bibr69-23312165261454531] Lütkenhöner B. Krumbholz K. Lammertmann C. Seither-Preisler A. Steinstrater O. Patterson R. D. (2003a). Localization of primary auditory cortex in humans by magnetoencephalography. Neuroimage, 18(1), 58–66. 10.1006/nimg.2002.1325 12507443

[bibr70-23312165261454531] Lütkenhöner B. Krumbholz K. Seither-Preisler A. (2003b). Studies of tonotopy based on wave N100 of the auditory evoked field are problematic. Neuroimage, 19(3), 935–949. 10.1016/s1053-8119(03)00172-1 12880822

[bibr71-23312165261454531] Mancinelli C. Livesu M. Puppo E. (2019). A comparison of methods for gradient field estimation on simplicial meshes. Computers & Graphics, 80, 37–50. 10.1016/j.cag.2019.03.005

[bibr72-23312165261454531] Marie D. Jobard G. Crivello F. Perchey G. Petit L. Mellet E. , …, & Tzourio-Mazoyer N. (2015). Descriptive anatomy of Heschl's gyri in 430 healthy volunteers, including 198 left-handers. Brain Structure & Function, 220(2), 729–743. 10.1007/s00429-013-0680-x 24310352 PMC4341020

[bibr73-23312165261454531] McCandless G. A. Best L. (1966). Summed evoked responses using pure-tone stimuli. Journal of Speech and Hearing Research, 9(2), 266–272. 10.1044/jshr.0902.266 5951219

[bibr74-23312165261454531] McFarland W. H. Vivion M. C. Goldstein R. (1977). Middle components of the AER to tone-pips in normal-hearing and hearing-impaired subjects. Journal of Speech and Hearing Research, 20(4), 781–798. 10.1044/jshr.2004.781 604690

[bibr75-23312165261454531] Micheyl C. Xiao L. Oxenham A. J. (2012). Characterizing the dependence of pure-tone frequency difference limens on frequency, duration, and level. Hearing Research, 292(1-2), 1–13. 10.1016/j.heares.2012.07.004 22841571 PMC3455123

[bibr76-23312165261454531] Mlynarski W. F. Hermundstad A. M. (2021). Efficient and adaptive sensory codes. Nature Neuroscience, 24(7), 998–1009. 10.1038/s41593-021-00846-0 34017131

[bibr77-23312165261454531] Moerel M. De Martino F. Formisano E. (2012). Processing of natural sounds in human auditory cortex: Tonotopy, spectral tuning, and relation to voice sensitivity. Journal of Neuroscience, 32(41), 14205–14216. 10.1523/JNEUROSCI.1388-12.2012 23055490 PMC6622378

[bibr78-23312165261454531] Moore B. C. (1986). Parallels between frequency selectivity measured psychophysically and in cochlear mechanics. Scandinavian Audiology. Supplementum, 25, 139–152. https://europepmc.org/article/MED/3472318 . 3472318

[bibr79-23312165261454531] Moore B. C. Glasberg B. R. Baer T. (1997). A model for the prediction of thresholds, loudness; and partial loudness. Journal of the Audio Engineering Society, 45(4), 224–240.

[bibr80-23312165261454531] Moore B. C. Glasberg B. R. Stone M. A. (2004). New version of the TEN test with calibrations in dB HL. Ear and Hearing, 25(5), 478–487. 10.1097/01.aud.0000145992.31135.89 15599194

[bibr81-23312165261454531] Morosan P. Rademacher J. Schleicher A. Amunts K. Schormann T. Zilles K. (2001). Human primary auditory cortex: Cytoarchitectonic subdivisions and mapping into a spatial reference system. Neuroimage, 13(4), 684–701. 10.1006/nimg.2000.0715 11305897

[bibr82-23312165261454531] Muir D. (2025). Compute a Tutte map of a planar surface triangulation: MATLAB central file exchange. https://www.mathworks.com/matlabcentral/fileexchange/32726-compute-a-tutte-map-of-a-planar-surface-triangulation.

[bibr83-23312165261454531] Musiek F. Nagle S. (2018). The middle latency response: A review of findings in various central nervous system lesions. Journal of the American Academy of Audiology, 29(9), 855–867. 10.3766/jaaa.16141 30278870

[bibr84-23312165261454531] Näätänen R. Picton T. (1987). The N1 wave of the human electric and magnetic response to sound: A review and an analysis of the component structure. Psychophysiology, 24(4), 375–425. 10.1111/j.1469-8986.1987.tb00311.x 3615753

[bibr85-23312165261454531] Naka D. Kakigi R. Hoshiyama M. Yamasaki H. Okusa T. Koyama S. (1999). Structure of the auditory evoked magnetic fields during sleep. Neuroscience, 93(2), 573–583. 10.1016/s0306-4522(99)00177-3 10465441

[bibr86-23312165261454531] Nasiotis K. Clavagnier S. Baillet S. Pack C. C. (2017). High-resolution retinotopic maps estimated with magnetoencephalography. Neuroimage, 145(Pt A), 107–117. 10.1016/j.neuroimage.2016.10.017 27743901

[bibr87-23312165261454531] Nunez P. L. Srinivasan R. (2006). Electric fields of the brain: The neurophysics of EEG. Oxford University Press. 10.1093/acprof:oso/9780195050387.001.0001

[bibr88-23312165261454531] Oates P. Stapells D. R. (1997). Frequency specificity of the human auditory brainstem and middle latency responses to brief tones. I. High-pass noise masking. Journal of the Acoustical Society of America, 102(6), 3597–3608. 10.1121/1.420148 9407653

[bibr89-23312165261454531] O'Neill G. C. Sengupta A. Asghar M. Barratt E. L. Besle J. Schluppeck D. , …, & Sanchez Panchuelo R. M. (2020). A probabilistic atlas of finger dominance in the primary somatosensory cortex. Neuroimage, 217, 116880. 10.1016/j.neuroimage.2020.116880 32376303 PMC7339146

[bibr90-23312165261454531] Pantev C. Bertrand O. Eulitz C. Verkindt C. Hampson S. Schuierer G. Elbert T. (1995). Specific tonotopic organizations of different areas of the human auditory cortex revealed by simultaneous magnetic and electric recordings. Electroencephalography and Clinical Neurophysiology, 94(1), 26–40. 10.1016/0013-4694(94)00209-4 7530637

[bibr91-23312165261454531] Pantev C. Roberts L. E. Elbert T. Ross B. Wienbruch C. (1996). Tonotopic organization of the sources of human auditory steady-state responses. Hearing Research, 101(1-2), 62–74. 10.1016/s0378-5955(96)00133-5 8951433

[bibr92-23312165261454531] Pascual-Marqui R. D. Michel C. M. Lehmann D. (1994). Low resolution electromagnetic tomography: A new method for localizing electrical activity in the brain. International Journal of Psychophysiology, 18(1), 49–65. 10.1016/0167-8760(84)90014-x 7876038

[bibr93-23312165261454531] Perez-Gonzalez D. Malmierca M. S. (2014). Adaptation in the auditory system: An overview. Frontiers in Integrative Neuroscience, 8, 19. 10.3389/fnint.2014.00019 24600361 PMC3931124

[bibr94-23312165261454531] Petkov C. I. Kayser C. Augath M. Logothetis N. K. (2006). Functional imaging reveals numerous fields in the monkey auditory cortex. PLoS Biology, 4(7), e215. 10.1371/journal.pbio.0040215 PMC1479693 16774452

[bibr95-23312165261454531] Picton T. W. (1988). The Endogenous Evoked Potentials. In Başar E. (Ed.), Dynamics of sensory and cognitive processing by the brain. Springer Series in Brain Dynamics, vol 1 (pp. 258–265). Springer. 10.1007/978-3-642-71531-0_17

[bibr96-23312165261454531] Picton T. W. (2010). Human auditory evoked potentials (1st ed.). Plural Publishing. ISBN: 978-1-59756-362-8.

[bibr97-23312165261454531] Picton T. W. John M. S. Dimitrijevic A. Purcell D. (2003). Human auditory steady-state responses. International Journal of Audiology, 42(4), 177–219. 10.3109/14992020309101316 12790346

[bibr98-23312165261454531] Picton T. W. Skinner C. R. Champagne S. C. Kellett A. J. Maiste A. C. (1987). Potentials evoked by the sinusoidal modulation of the amplitude or frequency of a tone. Journal of the Acoustical Society of America, 82(1), 165–178. 10.1121/1.395560 3624637

[bibr99-23312165261454531] Picton T. W. Woods D. L. Proulx G. B. (1978). Human auditory sustained potentials. II. Stimulus relationships. Electroencephalography and Clinical Neurophysiology, 45(2), 198–210. 10.1016/0013-4694(78)90004-4 78830

[bibr100-23312165261454531] R Core Team. (2025). R: A language and environment for statistical computing. R Foundation for Statistical Computing. https://www.R-project.org.

[bibr101-23312165261454531] Recanzone G. (2017) Auditory Processing in the Aging Brain. In Sherman S. M. (Ed.), Oxford research encyclopedia of neuroscience. Oxford University Press. 10.1093/acrefore/9780190264086.013.89

[bibr102-23312165261454531] Rodriguez R. Picton T. Linden D. Hamel G. Laframboise G. (1986). Human auditory steady state responses: Effects of intensity and frequency. Ear and Hearing, 7(5), 300–313. 10.1097/00003446-198610000-00003 3770325

[bibr103-23312165261454531] Rohatgi A. (2020, October 29). WebPlotDigitizer. https://automeris.io/WebPlotDigitizer.

[bibr104-23312165261454531] Rosburg T. Kreitschmann-Andermahr I. Emmerich E. Nowak H. Sauer H. (1998). Hemispheric differences in frequency dependent dipole orientation of the human auditory evoked field component N100 m. Neuroscience Letters, 258(2), 105–108. 10.1016/s0304-3940(98)00865-9 9875538

[bibr105-23312165261454531] Ross B. Borgmann C. Draganova R. Roberts L. E. Pantev C. (2000). A high-precision magnetoencephalographic study of human auditory steady-state responses to amplitude-modulated tones. Journal of the Acoustical Society of America, 108(2), 679–691. 10.1121/1.429600 10955634

[bibr106-23312165261454531] Ross B. Draganova R. Picton T. W. Pantev C. (2003). Frequency specificity of 40-hz auditory steady-state responses. Hearing Research, 186(1-2), 57–68. 10.1016/s0378-5955(03)00299-5 14644459

[bibr107-23312165261454531] Rothman H. H. (1970). Effects of high frequencies and intersubject variability on the auditory-evoked cortical response. Journal of the Acoustical Society of America, 47(2), 569–573. 10.1121/1.1911930 5439656

[bibr108-23312165261454531] Rouiller E. M. Durif C. (2004). The dual pattern of corticothalamic projection of the primary auditory cortex in macaque monkey. Neuroscience Letters, 358(1), 49–52. 10.1016/j.neulet.2004.01.008 15016432

[bibr500-23312165261454531] Rushworth C. Krumbholz K. (2026). Auditory-evoked response amplitude-frequency dependence: Meta-analysis and simulations. GitHub. https:/github.com/mszkk3/Auditory-evoked-response-amplitude-frequency-dependence-meta-analysis-and-simulations

[bibr109-23312165261454531] Saenz M. Langers D. R. (2014). Tonotopic mapping of human auditory cortex. Hearing Research, 307, 42–52. 10.1016/j.heares.2013.07.016 23916753

[bibr110-23312165261454531] Sanchez-Panchuelo R. M. Besle J. Beckett A. Bowtell R. Schluppeck D. Francis S. (2012). Within-digit functional parcellation of Brodmann areas of the human primary somatosensory cortex using functional magnetic resonance imaging at 7 tesla. Journal of Neuroscience, 32(45), 15815–15822. 10.1523/JNEUROSCI.2501-12.2012 23136420 PMC6621625

[bibr111-23312165261454531] Scherg M. Berg P. Nakasato N. Beniczky S. (2019). Taking the EEG back into the brain: The power of multiple discrete sources. Frontiers in Neurology, 10, 855. 10.3389/fneur.2019.00855 31481921 PMC6710389

[bibr112-23312165261454531] Scherg M. von Cramon D. (1985). Two bilateral sources of the late AEP as identified by a spatio-temporal dipole model. Electroencephalography and Clinical Neurophysiology, 62(1), 32–44. 10.1016/0168-5597(85)90033-4 2578376

[bibr113-23312165261454531] Scherg M. von Cramon D. (1986). Evoked dipole source potentials of the human auditory cortex. Electroencephalography and Clinical Neurophysiology, 65(5), 344–360. 10.1016/0168-5597(86)90014-6 2427326

[bibr114-23312165261454531] Schönwiesner M. Dechent P. Voit D. Petkov C. I. Krumbholz K. (2015). Parcellation of human and monkey core auditory Cortex with fMRI pattern classification and objective detection of tonotopic gradient reversals. Cerebral Cortex, 25(10), 3278–3289. 10.1093/cercor/bhu124 24904067 PMC4585487

[bibr115-23312165261454531] Seifritz E. Esposito F. Hennel F. Mustovic H. Neuhoff J. G. Bilecen D. , …, & Di Salle F. (2002). Spatiotemporal pattern of neural processing in the human auditory cortex. Science, 297(5587), 1706–1708. 10.1126/science.1074355 12215648

[bibr116-23312165261454531] Sereno M. I. Dale A. M. Reppas J. B. Kwong K. K. Belliveau J. W. Brady T. J. , …, & Tootell R. B. (1995). Borders of multiple visual areas in humans revealed by functional magnetic resonance imaging. Science, 268(5212), 889–893. 10.1126/science.7754376 7754376

[bibr117-23312165261454531] Sereno M. I. Lutti A. Weiskopf N. Dick F. (2013). Mapping the human cortical surface by combining quantitative T(1) with retinotopy. Cerebral Cortex, 23(9), 2261–2268. 10.1093/cercor/bhs213 22826609 PMC3729202

[bibr118-23312165261454531] Shams Z. Norris D. G. Marques J. P. (2019). A comparison of in vivo MRI based cortical myelin mapping using T1w/T2w and R1 mapping at 3 T. PLoS One, 14(7), e0218089. 10.1371/journal.pone.0218089 PMC6609014 31269041

[bibr119-23312165261454531] Shera C. A. Guinan J. J. Jr. Oxenham A. J. (2002). Revised estimates of human cochlear tuning from otoacoustic and behavioral measurements. Proceedings of the National Academy of Sciences of the United States of America, 99(5), 3318–3323. 10.1073/pnas.032675099 11867706 PMC122516

[bibr120-23312165261454531] Shmuel A. Yacoub E. Chaimow D. Logothetis N. K. Ugurbil K. (2007). Spatio-temporal point-spread function of fMRI signal in human gray matter at 7 tesla. Neuroimage, 35(2), 539–552. 10.1016/j.neuroimage.2006.12.030 17306989 PMC2989431

[bibr121-23312165261454531] Sigalovsky I. S. Fischl B. Melcher J. R. (2006). Mapping an intrinsic MR property of gray matter in auditory cortex of living humans: A possible marker for primary cortex and hemispheric differences. Neuroimage, 32(4), 1524–1537. 10.1016/j.neuroimage.2006.05.023 16806989 PMC1839042

[bibr122-23312165261454531] Stelmack R. M. Achorn E. Michaud A. (1977). Extraversion and individual differences in auditory evoked response. Psychophysiology, 14(4), 368–374. 10.1111/j.1469-8986.1977.tb02966.x 882616

[bibr123-23312165261454531] Striem-Amit E. Hertz U. Amedi A. (2011). Extensive cochleotopic mapping of human auditory cortical fields obtained with phase-encoding FMRI. PLoS One, 6(3), e17832. 10.1371/journal.pone.0017832 PMC3063163 21448274

[bibr124-23312165261454531] Sumner C. J. Wells T. T. Bergevin C. Sollini J. Kreft H. A. Palmer A. R. , …, & Shera C. A. (2018). Mammalian behavior and physiology converge to confirm sharper cochlear tuning in humans. Proceedings of the National Academy of Sciences of the United States of America, 115(44), 11322–11326. 10.1073/pnas.1810766115 30322908 PMC6217411

[bibr125-23312165261454531] Talavage T. M. Sereno M. I. Melcher J. R. Ledden P. J. Rosen B. R. Dale A. M. (2004). Tonotopic organization in human auditory cortex revealed by progressions of frequency sensitivity. Journal of Neurophysiology, 91(3), 1282–1296. 10.1152/jn.01125.2002 14614108

[bibr126-23312165261454531] Tecchio F. Bicciolo G. De Campora E. Pasqualetti P. Pizzella V. Indovina I. , …, & Rossini P. M. (2000). Tonotopic cortical changes following stapes substitution in otosclerotic patients: A magnetoencephalographic study. Human Brain Mapping, 10(1), 28–38. 10.1002/(sici)1097-0193(200005)10:1<28::aid-hbm40>3.0.co;2-g 10843516 PMC6871894

[bibr127-23312165261454531] Thomas J. M. Huber E. Stecker G. C. Boynton G. M. Saenz M. Fine I. (2015). Population receptive field estimates of human auditory cortex. Neuroimage, 105, 428–439. 10.1016/j.neuroimage.2014.10.060 25449742 PMC4262557

[bibr128-23312165261454531] Thornton A. R. Coleman M. J. (1975). The adaptation of cochlear and brainstem auditory evoked potentials in humans. Electroencephalography and Clinical Neurophysiology, 39(4), 399–406. 10.1016/0013-4694(75)90103-0 51723

[bibr129-23312165261454531] Thornton A. R. Mendel M. I. Anderson C. V. (1977). Effects of stimulus frequency and intensity on the middle componenets of the averaged auditory eletroencephalic response. Journal of Speech and Hearing Research, 20(1), 81–94. 10.1044/jshr.2001.81 846206

[bibr130-23312165261454531] Tiitinen H. Alho K. Huotilainen M. Ilmoniemi R. J. Simola J. Naatanen R. (1993). Tonotopic auditory cortex and the magnetoencephalographic (MEG) equivalent of the mismatch negativity. Psychophysiology, 30(5), 537–540. 10.1111/j.1469-8986.1993.tb02078.x 8416081

[bibr131-23312165261454531] Tlumak A. I. Durrant J. D. Delgado R. E. (2016). The effect of stimulus intensity and carrier frequency on auditory middle- and long-latency evoked potentials using a steady-state-response approach. American Journal of Audiology, 25(1), 62–74. 10.1044/2016_AJA-15-0061 26999323

[bibr132-23312165261454531] Tuomisto T. Hari R. Katila T. Poutanen T. Varpula T. (1983). Studies of auditory evoked magnetic and electric responses: Modality specificity and modelling. Il Nuovo Cimento D, 2(2), 471–483. 10.1007/BF02455946

[bibr133-23312165261454531] Tutte W. T. (1963). How to draw a graph. Proceedings of The London Mathematical Society, 13(1), 743–767. 10.1112/plms/s3-13.1.743

[bibr134-23312165261454531] Tzourio-Mazoyer N. Maingault S. Panzieri J. Pepe A. Crivello F. Mazoyer B. (2019). Intracortical myelination of Heschl’s gyrus and the planum temporale varies with Heschl’s duplication pattern and rhyming performance: An investigation of 440 healthy volunteers. Cerebral Cortex, 29(5), 2072–2083. https://doi.org/10.1093/cercor/bhy088 https://doi.org/10.1093/cercor/bhy088 29912300 10.1093/cercor/bhy088

[bibr135-23312165261454531] van de Moortele P. F. Auerbach E. J. Olman C. Yacoub E. Ugurbil K. Moeller S. (2009). T1 weighted brain images at 7 tesla unbiased for proton density, T2* contrast and RF coil receive B1 sensitivity with simultaneous vessel visualization. Neuroimage, 46(2), 432–446. 10.1016/j.neuroimage.2009.02.009 19233292 PMC2700263

[bibr136-23312165261454531] Vizioli L. Moeller S. Dowdle L. Akcakaya M. De Martino F. Yacoub E. Ugurbil K. (2021). Lowering the thermal noise barrier in functional brain mapping with magnetic resonance imaging. Nature Communications, 12(1), 5181. 10.1038/s41467-021-25431-8 PMC8405721 34462435

[bibr137-23312165261454531] Wallace M. N. Johnston P. W. Palmer A. R. (2002). Histochemical identification of cortical areas in the auditory region of the human brain. Experimental Brain Research, 143(4), 499–508. 10.1007/s00221-002-1014-z 11914796

[bibr138-23312165261454531] Wandell B. A. Dumoulin S. O. Brewer A. A. (2007). Visual field maps in human cortex. Neuron, 56(2), 366–383. 10.1016/j.neuron.2007.10.012 17964252

[bibr139-23312165261454531] Wandell B. A. Winawer J. (2011). Imaging retinotopic maps in the human brain. Vision Research, 51(7), 718–737. 10.1016/j.visres.2010.08.004 20692278 PMC3030662

[bibr140-23312165261454531] Warnking J. Dojat M. Guerin-Dugue A. Delon-Martin C. Olympieff S. Richard N. , …, & Segebarth C. (2002). fMRI retinotopic mapping–step by step. Neuroimage, 17(4), 1665–1683. 10.1006/nimg.2002.1304 12498741

[bibr141-23312165261454531] Zerlin S. Naunton R. F. Mowry H. J. (1973). The early evoked cortical response to third-octave clicks and tones. Audiology, 12(4), 242–249. https://www.ncbi.nlm.nih.gov/pubmed/4722023. https://doi.org/10.1080/00206097309089322 4722023

