# Supplementary material for: Reinterpreting the Frequency Dependence of Cortical Auditory-Evoked Response Amplitudes in Light of Current Understanding of Cortical Tonotopic Organization
Source: Trends Hear. 2026 Aug 4;30:23312165261454531. doi: 10.1177/23312165261454531 (PMC13438238; doi:10.1177/23312165261454531)

Supplementary Materials

FREQUENCY DEPENDENCE OF CORTICAL AER AMPLITUDES

Table S1: Literature search strings and results. The first column highlights each search string used; the second column indicates in which database the search was conducted (*Scholar* = Google Scholar, *PubMed* = U.S. National Library of Medicine's searchable bibliographic database; *NUSearch* = University of Nottingham Library's online search platform); and the third column (# of results) reports the total number of results returned prior to screening.

Search String	***Database***	***# of results***
"frequency specific" AND "auditory-evoked potential" AND human	*Scholar*	2,030
"frequency specific" AND "auditory-evoked potential" AND human	*Pubmed*	14
"frequency specific" AND "auditory-evoked potential" AND human	*NUSearch*	824
"frequency specific" AND "auditory-evoked field" AND human	*Scholar*	158
"frequency specific" AND "auditory-evoked field" AND human	*Pubmed*	1
"frequency specific" AND "auditory-evoked field" AND human	*NUSearch*	73
"frequency specific" AND "auditory-evoked response" AND human	*Scholar*	1190
"frequency specific" AND "auditory-evoked response" AND human	*Pubmed*	3
"frequency specific" AND "auditory-evoked response" AND human	*NUSearch*	357
"frequency specific" AND "vertex potential" AND human	*Scholar*	201
"frequency specific" AND "vertex potential" AND human	*Pubmed*	1
"frequency specific" AND "vertex potential" AND human	*NUSearch*	23
"stimulus frequency" AND "auditory-evoked potential" AND human	*Scholar*	1930
"stimulus frequency" AND "auditory-evoked potential" AND human	*Pubmed*	10
"stimulus frequency" AND "auditory-evoked potential" AND human	*NUSearch*	1065
"stimulus frequency" AND "auditory-evoked field" AND human	*Scholar*	221
"stimulus frequency" AND "auditory-evoked field" AND human	*Pubmed*	1
"stimulus frequency" AND "auditory-evoked field" AND human	*NUSearch*	111
"stimulus frequency" AND "auditory-evoked response" AND human	*Scholar*	1,160
"stimulus frequency" AND "auditory-evoked response" AND human	*Pubmed*	3
"stimulus frequency" AND "auditory-evoked response" AND human	*NUSearch*	428
"stimulus frequency" AND "vertex potential" AND human	*Scholar*	408
"stimulus frequency" AND "vertex potential" AND human	*Pubmed*	1
"stimulus frequency" AND "vertex potential" AND human	*NUSearch*	82

Figure S1: PRISMA diagram of literature search procedure. Adapted from Page *et al.* (2021), *The PRISMA 2020 statement: an updated guideline for reporting systematic reviews*, *BMJ*, 372:n71. The diagram indicates the total number of records identified in each database, the number of records screened, and the number of records remaining at each stage of the screening process. No automation tools were used, and all reports that passed title screening were successfully retrieved.

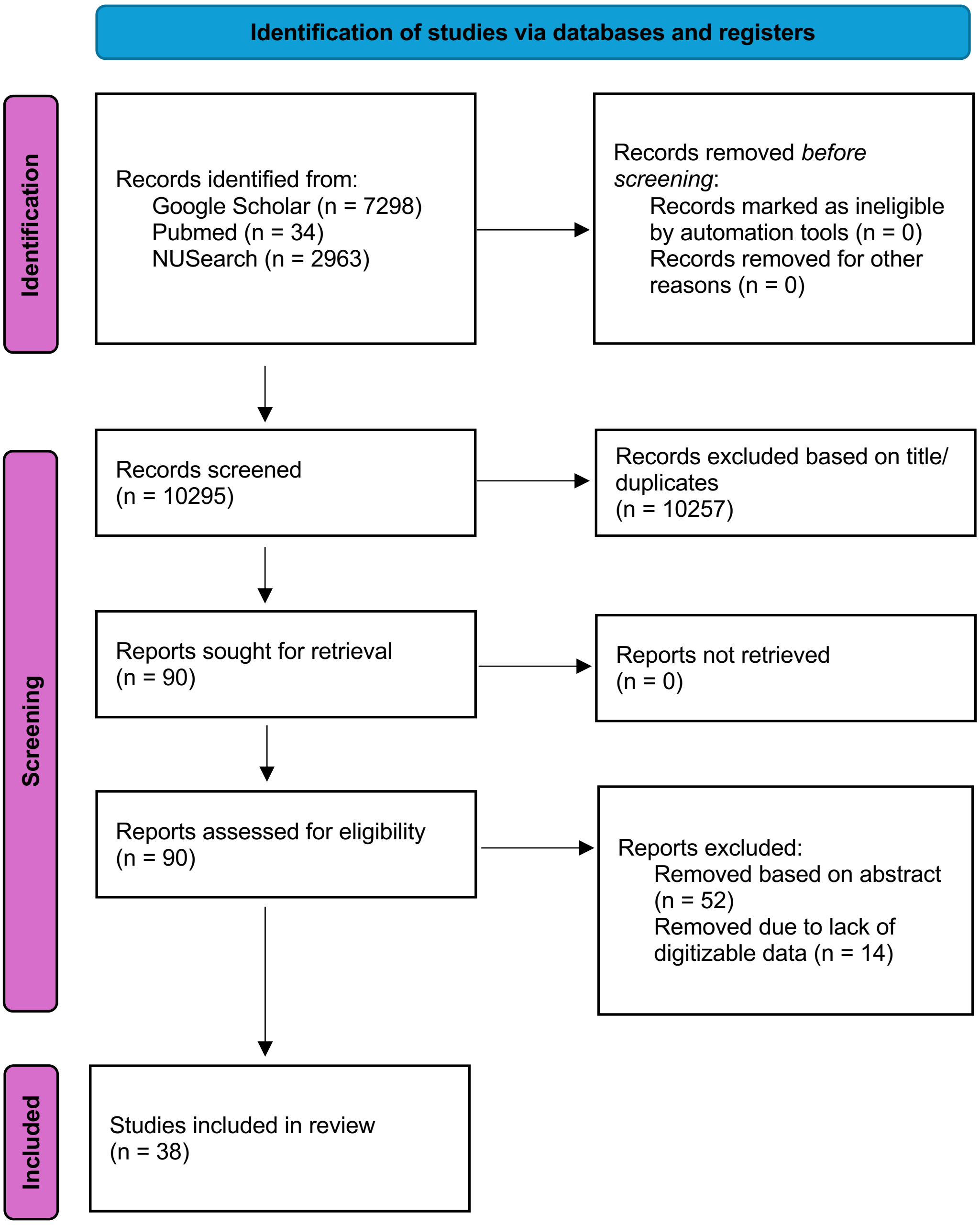

Table S2: Studies included in the meta-analysis of frequency-related decline in auditory-evoked response (AER) amplitude. The first column specifies the reported AER component ("m" in Pam and N1m stands for "magnetic"; **ABR** = auditory brainstem response; **ASSR** = auditory steady-state response; **MLR** = middle latency response; **LLR** = late-latency response). The frequency labels for steady-state responses ("40-Hz" and "80-Hz ASSR") are nominal. The third and fourth columns provide stimulus frequency and intensity information (HL = normal hearing level; SL = sensation level; SPL = sound pressure level; ppeSPL = peak-to-peak-equivalent SPL). The fifth column describes stimulus properties (mon. = monaural; bin. = binaural; contra. = contralateral to the sensor array; MM = mixed modulation; SAM/SFM = sinusoidal amplitude/frequency modulation). The sixth column provides the number of participants (N) included in a given study. Participant numbers are often approximate, particularly in older studies, many of which reported only overall sample sizes, while individual experiments within the same study often used smaller, undeclared subsets of participants (see main text for how N was derived). Finally, the seventh column specifies the recording modality (EEG = electroencephalography; MEG = magnetoencephalography). EEG studies used the vertical recording montage unless otherwise specified (vertex/Cz vs mastoid). For MEG studies, sensor type (FO = first-order; ax. = axial; grad. = gradiometer; mag. = magnetometer) and sensor arrangement (circular, linear, whole-head or single-channel) are indicated. References provided in the main text.

Component	Source	Frequencies (kHz)	Intensity levels	Stimulus information	N	Modality
**ABR** waves I, III & V	Eggermont and Don (1980), Fig. 3	0.5, 1, 2, 4, 8	10:10:60 dB HL	Derived-band clicks (mon.)	13	EEG
**ABR** 80-Hz ASSR	Dimitrijevic et al. (2002), Fig. 2	0.5, 1, 2, 4	5:5:35 dB SL	MM (100% SAM, 25% SFM; mon.)	14	EEG
**ABR** 80-Hz ASSR	John et al. (2001), Fig. 4	0.5, 0.75, 1, 2, 4, 1.5, 3, 6	30, 40, 50 dB SPL	100% SAM (mon.)	37	EEG
**ABR** 80-Hz ASSR	Lins et al. (1996), Fig. 5	0.5, 1, 2, 4	40, 50, 60 dB SPL	10% SAM (mon.)	40	EEG
**MLR** Pa-Nb	McFarland et al. (1977), Fig. 6	0.5, 1, 3	0, 10, 20, 35, 50 dB HL	6-ms tone pips (mon.)	10	EEG
**MLR** Pa-Nb	Oates and Stapells (1997), Fig. 2	0.5, 2	80 dB ppeSPL	2-1-2 tone pips (mon.)	12	EEG
**MLR** Pa-Nb	Thornton et al. (1977), Fig. 2	0.5, 1, 4	60, 70, 80 dB HL	6-ms tone pips (mon.)	11	EEG

**MLR** Pa-Nb	Zerlin et al. (1973), Fig. 3	0.5, 2	20, 50 dB SL	(i) 1/3-oct. filtered clicks (100 μs); (ii) 100-ms tone bursts (both bin.)	6	EEG
(i) **MLR** Pam (ii) **LLR** N1m	Pantev et al. (1995), Fig. 4	0.5, 1, 4	60 dB HL	50-ms tone bursts (contra.)	7	MEG (FO ax. grad.; circular)
40-Hz **ASSR**	Galambos et al. (1981), Fig. 3	0.25, 0.5, 2, 5	40 dB SL	6-ms tone bursts (mon.)	20	EEG
40-Hz **ASSR**	Kuwada et al. (1986), Fig. 5	.25, .5, 1, 2, 4, 8	20:20:100 dB SL	81% 50-Hz SAM (mon.)	1	EEG
40-Hz **ASSR**	Picton et al. (1987), Fig. 8	0.5, 1, 2	76.5 dB SPL	90% SAM, 50% SFM @ 31.3-46.9 Hz (mon.)	17	EEG
40-Hz **ASSR**	Rodriguez et al. (1986), Fig. 12	0.5, 2	10, 20, 30 dB SL	10-ms tone pips (mon.)	41	EEG
(i) 40-Hz **ASSR** (ii) **LLR***	Tlumak et al. (2016), Fig. 5	0.5, 1, 2	60 dB SPL	(i) 9.8-ms tone pips @ 40 Hz; (ii) 67.4-ms tone bursts @ 0.75 Hz (both mon.)	48	EEG
40-Hz **ASSR**	Pantev et al. (1996), Fig. 4	0.25, 0.5, 1, 2, 4	60 dB HL	5-ms (FWHM) Gaussian tone pips (contra.)	17	MEG (FO ax. grad.; circular)
40-Hz **ASSR**	Ross et al. (2000), Fig. 7	0.25, 0.5, 1, 2, 4	70 dB SL	100% SAM	8	MEG† (FO ax. grad.; circular)
40-Hz **ASSR**	Ross et al. (2003), Fig. 2	0.25, 0.5, 1, 2, 4	70 dB SL	100% SAM	6	MEG† (FO ax. grad.; circular)

**LLR** N1-P2	Antinoro et al. (1969), Fig. 2	0.125, 0.5, 1, 2, 4, 8	20:20:100 dB SL	75-ms tone bursts	6	EEG
**LLR** N1-P2	Davis et al. (1968), Fig. 5	0.25, 1, 4	30:30:90 dB HL	60-ms tone bursts (bin.)	3	EEG
**LLR** N1-P2	Evans and Deatherage (1969), Fig. 1	0.5, 1, 2, 4	45, 90 dB HL	200-ms tone bursts	4	EEG
**LLR** N1-P2	Guiraud et al. (2007), Fig. 2; Data from Verkindt et al. (1995)	0.5, 1, 2, 4	65 dB SL	200-ms tone bursts (mon.)	9	EEG
**LLR** N1	Jacobson et al. (1992), Table 3	0.25, 1, 4	60 dB SL	250-ms tone bursts (min.)	11	EEG (avg. Cz & Fz vs "linked mandibles")
**LLR** N1-P2	Lenhardt (1971), Fig. 1	0.5, 2, 4	10-dB SL increment from 60 dB SL	300-ms increments in continuous tone (mon.)	4	EEG
**LLR** N1-P2	McCandless and Best (1966), Fig.3	0.25, 1, 4	10, 30, 50 dB SL	700-ms tone bursts	10	EEG
**LLR** N1-P2	Picton et al. (1978), Fig. 6	0.25, 0.5, 1, 2, 4, 8	87 dB SPL	1-s tone bursts (mon.)	10	EEG
**LLR** N1-P2	Rothman (1970), Figs. 2,3	2, 4, 6, 8	10:20:70 dB SL	200-ms tone bursts (mon.)	6	EEG
**LLR** N1-P2	Stelmack et al. (1977), Figs. 1,3,4	0.5, 8	40, 55, 80 dBA	200-ms tone bursts (bin.)	30	EEG
(i) **LLR** N1-P2<br>(ii) **LLR** N1m	Tuomisto et al. (1983), Figs. 8,10	0.44, 1, 2.5, 8	40 dB SL	500-ms tone bursts (mon.)	1	(i) EEG; (ii) MEG (single FO grad.)

**LLR** N1m	Arlinger et al. (1982), Table 4	0.5, 1, 2, 4	60 dB SL	20% frequency glides with 20-ms glide duration (contra.)	3	MEG (FO ax. grad.; linear)
**LLR** N1m	Elberling et al. (1982), Fig. 2	0.5, 4	80 dB SPL	500-ms tone bursts (contra.)	4	MEG (FO ax. grad.; linear)
**LLR** N1m	Fujioka et al. (2002), Table 1	4, 6, 10, 12, 14	60 dB SPL	70-ms tone bursts (bin.)	4	MEG (mag.; dual-circular)
**LLR** N1m	Gabriel et al. (2004), Table 2	1, 2, 4, 6, 8, 12	60 dB SL	200-ms tone bursts (ipsi. & contra.)	6	MEG (mag.; whole-head)
**LLR** N1m	Lütkenhöner et al. (2003), Fig. 4	0.5, 1, 2, 4	60 dB SL	500-ms tone bursts (contra.)	10	MEG (FO ax. grad.; circular)
**LLR** N1m	Naka et al. (1999), Table 2	0.25, 1, 4	70 dB SPL	70-ms tone bursts (contra. with noise masker in opposite ear)	10	MEG (mag.; dual-circular)
**LLR** N1m	Rosburg et al. (1998), Table 1	1, 5	90 dB SPL	50-ms tone bursts (contra.)	17	MEG (mag.; near-planar hexagonal)
**LLR** N1m	Tecchio et al. (2000), Fig. 1	0.25, 0.25, 1, 2	50 dB SL	300-ms tone bursts (contra. with noise masker in opposite ear)	1	MEG (FO ax. grad. & mag.; circular)

*Tlumak et al. (2016) measured the size of the LLR in the frequency domain by calculating the harmonic sum (square root of sum of magnitudes) of response frequency components.

† Ross et al. (2000, 2003) measured the source strength of their responses in nAm. Their data are nonetheless included, because an average dipole location was used across frequencies.

Table S3: Component-specific effects of frequency. Results of likelihood ratio tests (df = 1) of the linear (*lgfrq*) and quadratic (*lgfrq*^{2}) fixed-effects frequency terms in the generalized linear mixed-effects (GLME) model analyses of the meta-analytic data for each AER component (ABR, MLR, LLR and 40-Hz ASSR). Section A displays results from unweighted model estimations, while Section B displays weighted model estimation results, where studies were weighted by the number of subjects included in each study (column labelled "*N*" in Table S1). Next to the parameter estimates (β) and 95% confidence intervals (95% CI) of *lgfrq* and *lgfrq*^{2}, we report the chi-squared statistic (χ^2) and its degrees of freedom (*df*), raw p-values (*p*), Holm-Bonferroni (HB) corrected p-values (p_{HB}, corrected for multiple comparisons across four AER components), and Bayes Factors (*BF*). Confidence intervals were derived using 95% semi-parametric *wild* bootstrap percentiles (5,000 resamples). Significant terms are highlighted in bold. The blue italic font highlights the parameter estimates (β-values) of the linear frequency term (expressed in decibel per octave) of the cortical transient-evoked (MLR, LLR) and steady-state (40-Hz ASSR) components.

A. Unweighted

Comp	Term	β	95% CI	χ^2	*df*	*p*	p_{HB}	*BF*
ABR	*lg2frq*	0.73	**[0.35, 1.11]**	7.01	1	.008	**.008**	**2.73**
	lg2frq^2	−0.72	**[−1.04, −0.40]**	8.94	1	.003	**.008**	**7.15**
MLR	*lg2frq*	*−0.37*	**[−0.50, −0.24]**	8.22	1	.004	**.008**	**10**
	lg2frq^2	−0.09	[−0.20, 0.01]	0.92	1	.338	.338	1/3.84
LLR	*lg2frq*	*−0.49*	**[−0.60, −0.38]**	26.84	1	<.001	**<.001**	**>150**
	lg2frq^2	−0.07	**[−0.12, −0.02]**	8.56	1	.003	**.008**	**6.14**
40-Hz ASSR	*lg2frq*	*−1.35*	**[−1.79, −0.90]**	12.57	1	<.001	**.001**	**64.3**
	lg2frq^2	−0.15	**[−0.19, −0.12]**	14.22	1	<.001	**<.001**	**146**

B. Weighted by number of study subjects (N)

Comp	Term	β	95% CI	χ^2	*df*	*p*	p_{HB}	*BF*
ABR	*lg2frq*	0.67	**[0.27, 1.08]**	6.21	1	.013	**.015**	**1.83**
	lg2frq^2	−0.72	**[−0.98, −0.45]**	10.14	1	.001	**.005**	**13**
MLR	*lg2frq*	*−0.35*	**[−0.45, −0.25]**	7.21	1	.007	**.015**	**6.04**
	lg2frq^2	−0.09	[−0.18, 0.00]	0.97	1	.324	.324	1/3.74
LLR	*lg2frq*	*−0.50*	**[−0.62, −0.39]**	25.95	1	<.001	**<.001**	**>150**
	lg2frq^2	−0.07	**[−0.12, −0.01]**	6.50	1	.011	**.022**	**2.19**
40-Hz ASSR	*lg2frq*	*−1.38*	**[−1.92, −0.82]**	11.93	1	<.001	**.002**	**46.5**
	lg2frq^2	−0.19	**[−0.32, −0.07]**	10.24	1	.001	**.005**	**20**

Table S4: Pairwise comparisons of frequency-amplitude slopes between ABRs and cortical AER components. Results of likelihood ratio tests evaluating differences in frequency-amplitude slopes between ABRs and cortical AER components (MLR, LLR and 40-Hz ASSR). The slope difference is represented by the interaction, *lgfrq*:*comp*, between the linear frequency term (*lgfrq*) and the component factor (*comp*). Section A shows results from unweighted model estimations, while Section B shows results from weighted model estimations based on the number of subjects (*N*). Statistics include the parameter estimate (β) and its 95% confidence interval (95% CI; *wild* bootstrapping with 5000 resamples), the chi-squared statistic (χ^2) and its degrees of freedom (*df*), raw p-value (*p*), Holm-Bonferroni (HB) corrected p-value (p_{HB}, corrected across three cortical AER components), and Bayes Factor (*BF*) for each model.

A. Unweighted

Model	β	95% CI	χ^2	*df*	*p*	p_{HB}	*BF*
ABR/MLR	–1.10	**[–1.51, –0.69]**	11.88	1	<.001	**<.001**	**27.9**
ABR/LLR	–1.29	**[–1.69, –0.89]**	23.73	1	<.001	**<.001**	**>150**
ABR/ASSR	–1.91	**[–2.66, –1.16]**	18.29	1	<.001	**<.001**	**>150**

B. Weighted by number of study subjects (N)

Model	β	95% CI	χ^2	*df*	*p*	p_{HB}	*BF*
ABR/MLR	–1.02	**[–1.46, –0.60]**	9.45	1	.002	**.002**	**8.26**
ABR/LLR	–1.18	**[–1.63, –0.73]**	17.49	1	<.001	**<.001**	**>150**
ABR/ASSR	–2.04	**[–2.93, –1.11]**	18.09	1	<.001	**<.001**	**>150**

Table S5: Omnibus comparison of frequency-amplitude slopes among cortical AER components. Likelihood ratio tests evaluating differences in frequency-amplitude slopes among cortical AER components (MLR, LLR and 40-Hz ASSR). As in Table S3, slope differences are represented by the interaction between the linear frequency term and the component factor (*lgfrq*:*comp*). The GLME model was evaluated either across all three cortical components (MLR/LLR/ASSR) or restricted to the transient-evoked components (MLR/LLR). The non-significance of the interaction in the latter model (excluding the ASSR; with BFs <1) demonstrates that the significance in the former (including the ASSR) was driven by the ASSR component. As in the Tables S2 and S3, results are provided for both unweighted (Section A) and weighted (Section B) model estimations. Statistics include parameter estimates (β), 95% confidence intervals (95% CI; *wild* bootstrapping with 5000 resamples), chi-squared statistics (χ^2) and associated degrees of freedom (*df*), raw p-values (*p*), and Bayes Factors (*BF*). In the case of the full model (MLR/LLR/ASSR), two parameter estimates are given, representing the slope differences between each of the two transient-evoked components (MLR, LLR) and the ASSR. The parameter estimate for the MLR/LLR model represents the slope for the MLR relative to that of the LLR.

A. Unweighted

Model	Contrast	β	95% CI	χ^2	*df*	*p*	*BF*
Full	MLR-ASSR	0.99	**[0.49, 1.48]**	24.01	2	**<.001**	**>150**
	LLR-ASSR	0.90	**[0.46, 1.33]**				
excl. ASSR	MLR-LLR	0.11	[–0.12, 0.34]	1.91	1	.167	1/5.11

B. Weighted by number of study subjects (N)

Model	*Contrast*	β	*95% CI*	χ^2	*df*	*p*	*BF*
Full	MLR-ASSR	0.94	**[0.38, 1.52]**	18.09	2	**<.001**	**34.5**
	LLR-ASSR	0.77	**[0.29, 1.26]**				
excl. ASSR	MLR-LLR	0.13	[–0.07, 0.32]	1.80	1	.180	1/5.41

Table S6: Comparison of the shape of the frequency-amplitude dependence of the LLR between EEG and MEG studies. Likelihood ratio test results evaluating differences in the shape of the frequency-amplitude dependence of the LLR between EEG and MEG studies. The slope difference is represented by the interactions between the linear and quadratic frequency terms (*lgfrq and* $lgfrq^2$) on the one hand, and measurement modality (*modl*) on the other. Results are provided for both the unweighted (Section A) and weighted (Section B) model estimation. Statistics include the parameter estimate (β), 95% confidence interval (*95% CI*; *wild* bootstrapping with 5000 resamples), chi-squared statistic (χ^2) and associated degrees of freedom (*df*), raw p-value (*p*), and Bayes Factor (*BF*) for each model. The interaction between modality and the quadratic frequency term reached significance in the unweighted model. In the weighted model, the likelihood ratio indicated a significant difference (bold font), but the Bayes factor provided weak evidence in favour of the null model (italic font).

A. Unweighted

Term	β	95% CI	χ^2	*df*	*p*	*BF*
lg2frq:*modl*	0.14	[–0.10, 0.38]	1.60	1	.206	1/5.29
$lg2frq^2$:*modl*	–0.14	**[–0.22, –0.06]**	6.61	1	**.010**	**2.31**

B. Weighted by number of study subjects (N)

Term	β	95% CI	χ^2	*df*	*p*	*BF*
lg2frq:*modl*	0.03	[–0.21, 0.26]	0.06	1	.812	1/11.5
$lg2frq^2$:*modl*	–0.13	**[–0.23, –0.04]**	4.61	1	**.032**	*1/1.18*

Table S7: Statistical results for semi-quantitative R1 values (A) and pRF tuning widths (B). Likelihood ratio tests (LRTs) and associated post-hoc comparisons of the effect of cortical area (A1, LP, PA) on area-averaged vertexwise longitudinal relaxation rate, R1 (Section A), and population receptive field (pRF) full width at half maximum (FWHM; Section B). The upper part of each table reports the LRT results. Statistics include parameter estimates (β) with semi-parametric bootstrap 95% confidence intervals (*wild* bootstrap, 5000 resamples), as well as the chi-squared statistic (χ^2) and associated degrees of freedom, p-value (p) and Bayes factor (BF). The lower part of each table reports the results of paired-samples t-tests between all three pairs of cortical areas (A1–LP, LP–PA, A1–PA), including both uncorrected p-values (p) and Holm–Bonferroni-corrected p-values (p_{HB}).

A. Semi-quantitative R1 values

Test	***Contrast***	***Statistics***
LRT	*LP-A1*	β = –0.10 CI [–0.12, –0.07]; $\chi^2(2)$ = 42.79; p < .001; BF > 150
	PA-A1	β = –0.10 CI [–0.13, –0.08]
t-test	*A1-LP*	t(15) = 8.25, p/p_c < .001/.001 → H = 1
	A1-PA	t(15) = 7.45, p/p_c < .001/.001 → H = 1
	LP-PA	t(15) = 0.55, p/p_c = .593/.593 → H = 0

B. pRF tuning widths

Test	***Contrast***	***Statistics***
LRT	*LP-A1*	β = 3.32 CI [0.15, 6.47]; $\chi^2(2)$ = 36.10; p < .001; BF > 150
	PA-A1	β = 13.34 CI [8.04, 18.61]
post-hoc	*A1-LP*	t(25) = –2.02, p/p_c = .055/.055 → H = 0
	A1-PA	t(23) = –5.73, p/p_c < .001/.001 → H = 1
	LP-PA	t(24) = –4.27, p/p_c < .001/.001 → H = 0

Figure S2: Effect of recording reference on simulated AER amplitudes. Simulated EEG amplitudes for the vertex (Cz) channel referenced to the linked mastoid electrodes (TP9 and TP10; black solid lines) or the average reference across all channels (yellow dashed lines). Simulation conditions were the same as for the ERB model shown in Fig. 8 of the main text (ERB tonotopy model, pRF-based neuronal tuning widths. As in Fig. 8, different panels show responses from different functional areas (A1, LP and PA) and the coloured lines and shading show the observed amplitude-frequency relationships for the MLR, LLR and 40-Hz ASSR derived from the meta-analytic data (replotted from Fig. 5). The close correspondence between the two simulated reference schemes indicates that the predicted frequency dependence of the vertex channel is largely insensitive to common referencing choices.

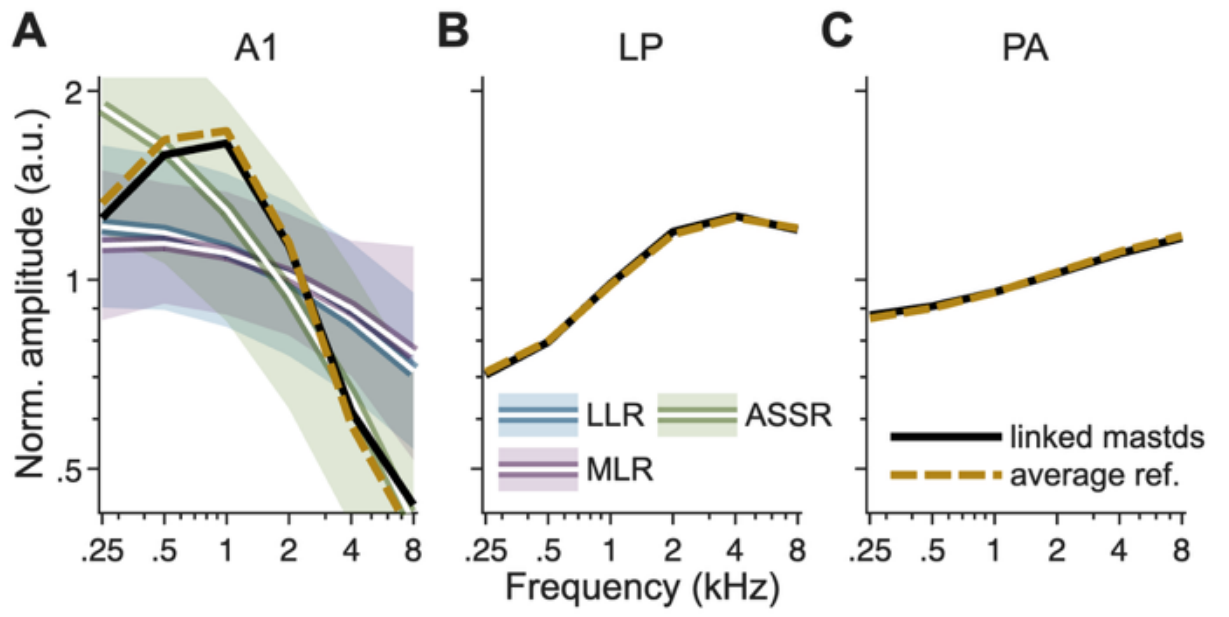

Supplement: Supplementary file 1 [file sj-pdf-1-tia-10.1177_23312165261454531.pdf]
